# NAT10-mediated ac4C modifications regulate glioblastoma progression

**DOI:** 10.1038/s41419-025-08315-3

**Published:** 2026-01-08

**Authors:** Li Lin, Yu Xiong, Yun Guo, Zewei Tu, PengXiang Luo, Zhansheng Fang, Longbo Zhang, Kai Huang, Lei Wu

**Affiliations:** 1 Department of Neurosurgery, The Second Affiliated Hospital of Nanchang University, Nanchang, Jiangxi China; 2 Institute of Neuroscience, Nanchang University, Nanchang, Jiangxi China; 3 Jiangxi Key Laboratory of Neurological Tumors and Cerebrovascular Diseases, Nanchang, Jiangxi China; 4 Department of Ophthalmology, The Second Affiliated Hospital of Nanchang University, Nanchang, Jiangxi China; 5 Department of Neurosurgery, National Clinical Research Center of Geriatric Disorders, Research Center for Cerebrovascular Disease, Xiangya Hospital, Central South University, Changsha, China

**Keywords:** RNA modification, CNS cancer, Transcriptional regulatory elements, Mechanisms of disease

## Abstract

N4-acetylcytidine (ac4C) is a recently identified mRNA modification, with N-acetyltransferase 10 (NAT10) being the sole known enzyme responsible for its catalysis. However, the biological functions and regulatory mechanisms of NAT10-mediated ac4C modification in glioblastoma (GBM) remain largely unclear. In this study, we aimed to elucidate the regulatory pathways and functional implications of NAT10 and ac4C modification in GBM. We found that NAT10 is significantly upregulated in GBM, and its elevated expression is associated with disease progression and poor patient prognosis. Functionally, NAT10 promotes glioblastoma cell proliferation and migration in vitro and accelerates tumor growth in vivo. Mechanistically, we identified BOC mRNA, a member of the immunoglobulin superfamily of cell adhesion molecules, as a direct target of NAT10-catalyzed ac4C modification. This modification enhances both the stability and translational efficiency of BOC mRNA, thereby contributing to GBM progression. Furthermore, we demonstrate that HIF1α, a key transcription factor in the hypoxic response, directly activates NAT10 transcription by binding to hypoxia response elements HRE1 and HRE2, leading to increased ac4C modification of BOC mRNA under hypoxic conditions. Notably, pharmacological inhibition of NAT10 effectively suppresses its enzymatic activity, particularly under hypoxia, underscoring its potential as a therapeutic target in GBM. In summary, our findings reveal a critical role for NAT10-mediated mRNA ac4C modification in GBM oncogenesis and highlight NAT10 as a promising target for therapeutic intervention.

**Figure:**
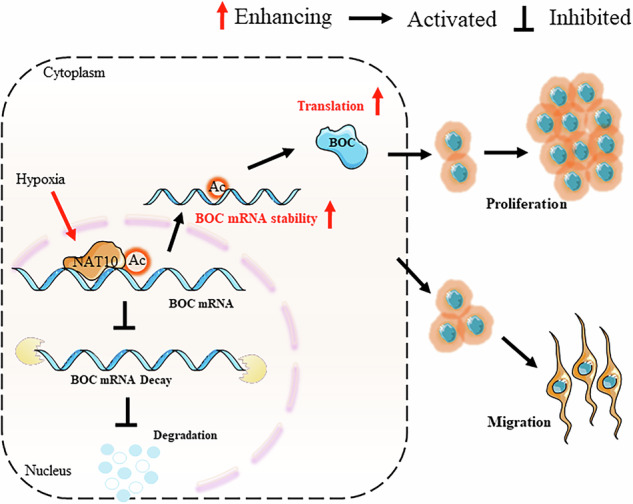
NAT10 was upregulated in GBM, and NAT10 facilitated GBM progression in vitro and in vivo. Mechanistically, NAT10 catalyzed ac4C modification of BOC mRNA and maintained its stability and promoted translation. Besides, HIF1α influenced NAT10 and its ac4C writer function through transcriptional activation.

NAT10 was upregulated in GBM, and NAT10 facilitated GBM progression in vitro and in vivo. Mechanistically, NAT10 catalyzed ac4C modification of BOC mRNA and maintained its stability and promoted translation. Besides, HIF1α influenced NAT10 and its ac4C writer function through transcriptional activation.

## Introduction

Glioblastoma (GBM) is the most common and aggressive primary malignancy of the central nervous system in adults [1]. Characterized by rapid proliferation, diffuse infiltration, and resistance to conventional therapies, GBM remains a formidable clinical challenge. Despite advances in surgical resection, radiotherapy, and chemotherapy, patient outcomes remain dismal, with a median survival of approximately 15 months post-diagnosis. The advent of tumor-treating fields (TTFields), a novel therapeutic modality that employs alternating electric fields to disrupt mitotic processes in tumor cells, has introduced a new avenue for treating both newly diagnosed and recurrent GBM [2]. Clinical trials have shown that TTFields can modestly improve both progression-free survival and overall survival (OS) by 2.7 and 4.9 months, respectively [3]. However, the clinical benefits of TTFields are accompanied by considerable financial burden, underscoring the urgent need to explore more effective and accessible therapeutic strategies for GBM.

Current investigations into GBM focus on several critical aspects, including tumor heterogeneity, epigenetic regulation, immune evasion, and the tumor microenvironment [4, 5]. Among these, epigenetic modifications, such as DNA methylation and histone alterations, have been shown to regulate gene expression and contribute to tumor initiation and progression [6]. In recent years, a growing body of research has uncovered an additional regulatory layer: RNA modifications, which have emerged as important players in cancer biology. Among them, N4-acetylcytidine (ac4C) has gained particular attention for its role in post-transcriptional gene regulation [7]. ac4C is a chemical modification that occurs on cytidine residues in mRNA and primarily catalyzed by N-acetyltransferase 10 (NAT10)-currently the only known ac4C “writer” enzyme in eukaryotes [8]. This modification enhances mRNA stability and translation efficiency, ultimately impacting protein expression and cellular behavior. Several studies have demonstrated the oncogenic potential of NAT10-mediated ac4C modification across different cancer types. For instance, NAT10 promotes metastasis in esophageal cancer by increasing NOTCH3 mRNA stability in an ac4C-dependent manner [9]. Similarly, in cervical cancer, NAT10 stimulates ac4C modification of FOXP1 mRNA, enhancing its translation and leading to increased glycolysis and immunosuppression [10]. Moreover, in bladder cancer, NAT10 has been shown to drive cisplatin resistance by promoting ac4C-associated DNA repair pathways [11]. Collectively, these findings highlight the importance of RNA modifications, particularly ac4C, in regulating oncogenic processes. Dysregulation of such modifications can result in aberrant gene expression, thereby contributing to the development and progression of a wide range of cancers, including GBM.

This study aims to elucidate the role of NAT10 in GBM by investigating its expression patterns, regulatory mechanisms, and functional impact on mRNA substrates, particularly BOC mRNA. BOC is a cell surface receptor of the immunoglobulin superfamily that plays a critical role in Sonic Hedgehog (SHH)-dependent axon guidance, essential for proper neural circuit formation in the nervous system [12–14]. In the context of GBM, previous studies have reported that BOC promotes tumor progression by targeting Smoothened to enhance SHH signaling, thereby driving glioma cell proliferation, migration, and invasion. Its role in medulloblastoma progression further underscores its importance in brain tumor development [15, 16]. In our study, we demonstrated that NAT10 was significantly overexpressed in GBM tissues compared to normal brain tissue, and this elevated expression correlated with poor patient prognosis. We identified BOC mRNA as a key downstream substrate of NAT10, and showed that NAT10-mediated ac4C modification enhanced the stability and translational efficiency of BOC mRNA. This post-transcriptional regulation ultimately contributed to the malignant phenotype of GBM cells. Furthermore, we explored how the tumor microenvironment, particularly hypoxia, influenced NAT10 expression and ac4C catalytic activity. Hypoxia, a common feature of solid tumors, is known to regulate various cellular behaviors, including proliferation, survival, and invasiveness [17]. Our findings revealed that hypoxic stress upregulates NAT10 expression and enhances its activity, thereby amplifying ac4C-mediated mRNA modifications in GBM. Collectively, our study provided novel insights into the epitranscriptomic regulation of GBM through NAT10-mediated ac4C modification and highlighted the dynamic interplay between the tumor microenvironment and RNA modification machinery. These findings offered potential avenues for developing targeted therapeutic strategies against GBM.

## Results

### NAT10 expression was elevated in GBM and correlated with poor patient prognosis

**Fig. 1:**
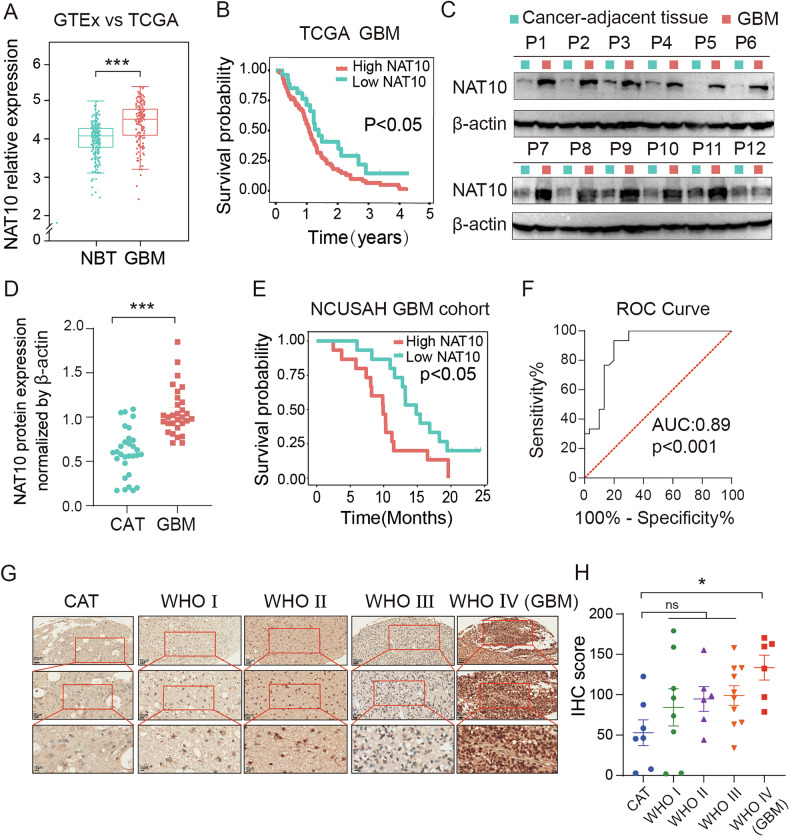
NAT10 was extremely upregulated and correlated with worse prognosis of GBM. **A** The levels of *NAT10* between normal brain tissues (NBT) and GBM group. **B** Kaplan-Meier survival curve of patients with GBM in the TCGA databases. **C**, **D** The expression of NAT10 was detected by Western blot (WB) in glioma and cancer adjacent tissues (CAT) (*n* = 30). **E** Kaplan-Meier survival curve of patients with GBM in the Neurosurgery Department of the Second Affiliated Hospital of Nanchang University (NCUSAH) databases. **F** The ROC model to distinguish GBM patients and healthy individuals. **G** IHC staining verified that NAT10 protein level was elevated in GBM patient samples **H**. T-test was applied to compare the IHC-score of NAT10 between CAT and GBM samples. ns *p* > 0.05, *p < 0.05, **p < 0.01, ***p < 0.001.

N-acetyltransferase 10 (NAT10) is a highly conserved protein across vertebrate species, distinguished by its N-acetyltransferase domain. It is the sole known regulator of mRNA acetylation and has been implicated in various malignancies [18]. To investigate the tumor-specific expression profile of *NAT10*, we analyzed gene expression profiling (GEP) data across 33 tumor types using the SangerBox database (http://vip.sangerbox.com/home.html) [19]. Our analysis revealed that *NAT10* was significantly upregulated in a majority of tumors, with particularly high expression observed in glioblastomas (GBMs) **(**Figs. S1A, 1A**)**. Further, *NAT10* expression was more abundance in high-grade gliomas (HGGs) compared to low-grade gliomas (LGGs) across three independent datasets, TCGA, CGGA, and GSE108474 **(**Fig. S1B**)**. Kaplan–Meier survival analyses using these datasets showed that elevated *NAT10* expression correlated with poorer overall survival (OS) in GBM patients, as well as across general glioma cohorts **(**Fig. 1B, S1C–G**)**. To validate these findings at the protein level, we examined 30 paired GBM and cancer-adjacent tissue (CAT) samples. Immunoblotting revealed markedly higher NAT10 protein levels in tumor tissues compared to adjacent tissues **(**Figs. 1C, D, S1H**)**. Survival analysis also indicated that patients with higher *NAT10* levels had significantly shorter survival times and lower OS rates **(**Fig. 1E**)**. Receiver Operating Characteristic (ROC) curve analysis suggested that *NAT10* has discriminative power as a potential diagnostic biomarker, distinguishing GBM tissues from non-tumor tissues **(**Fig. 1F**)**. Additionally, analysis of a commercial glioma tissue microarray, including six GBM sections, five adjacent tissues, two normal brain samples, and 26 non-GBM tumor tissues, confirmed the overexpression of NAT10 in GBM via IHC staining **(**Fig. 1G–H**)**. Collectively, these results demonstrated that NAT10 was significantly overexpressed in GBM at both the mRNA and protein levels, correlated with poor prognosis, and may played a critical role in the malignant progression of glioblastoma.

### Inhibition of GBM tumor progression through NAT10 disruption

**Fig. 2:**
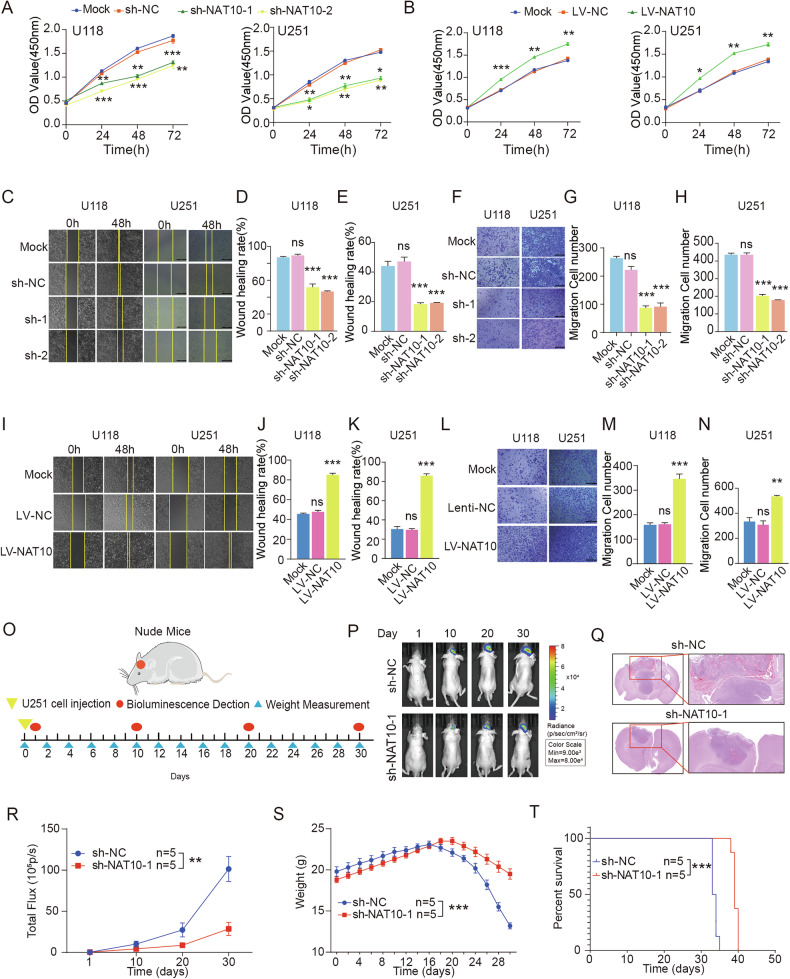
The deletion of NAT10 restrained GBM growth, and migration in vivo and in vitro. **A**–**N** The CCK8 assay, Transwell migration assay, and Wound healing assay to detect the proliferation and migration ability of NAT10-knockdown and overexpress stably transfected U118 and U251 cells. **O** The workflow of animal model study. **P** Continuous tumor fluorescence intensities were determined in vivo by using an IVIS Lumina XR Series III image system at 1, 10, 20, and 30 days. **Q** Representative HE staining images provided a visual representation of the tumor size in each xenograft group. Scale bars, 200 μm. **R** The total flux at each time points dynamically reflected tumor size changes in each nude mice group. **S** The line chart depicted the variation in nude mice weight across the groups. **T** Kaplan–Meier survival analysis demonstrated the distinct prognosis of each group. ns *p* > 0.05, *p < 0.05, **p < 0.01, ***p < 0.001.

To comprehensively investigate the functional role of NAT10 in GBM, we first assessed its protein levels across five GBM cell lines in comparison to human astrocyte (HA) cells. Most GBM lines exhibited elevated NAT10 expression relative to HA cells. Among them, U118 and U251 showed the highest expression **(**Fig. S1I**)**, and were therefore selected for subsequent functional studies. We generated NAT10-knockdown (sh-NAT10) and NAT10-overexpressing (LV-NAT10) models in both cell lines, with successful modulation confirmed by WB **(**Fig. S2A, B**)**. Cell proliferation assays (CCK-8) revealed that NAT10 knockdown significantly reduced proliferation, while NAT10 overexpression enhanced it, compared to mock and negative control groups **(**Fig. 2A, B**)**. In addition, scratch wound healing and transwell migration assays demonstrated that NAT10 silencing suppressed the migration of GBM cells, whereas its overexpression promoted it **(**Fig. 2C–N**)**. Given the importance of epithelial–mesenchymal transition (EMT) in malignant progression [20], we next assessed the impact of NAT10 on EMT-associated cellular morphology. NAT10 knockdown resulted in a marked increase in the epithelial marker E-cadherin, alongside decreased expression of the mesenchymal markers N-cadherin and Vimentin, as well as the proliferation marker PCNA **(**Fig. S2C–H**)**. Immunofluorescence assays confirmed that silencing NAT10 inhibited EMT markers in GBM cells, which was demonstrated by the upregulation of E-cadherin and downregulation of N-cadherin and vimentin **(**Fig, S2I**)**. Consistent with these molecular alterations, microscopic observation of cell morphology showed that control cells exhibited an elongated, spindle-shaped morphology, whereas NAT10-depleted cells acquired a compact, cobblestone-like epithelial shape, providing direct visual evidence for the suppression of EMT **(**Fig. S2I**)**. These results suggested that NAT10 promoted the mesenchymal phenotype and facilitated GBM cell proliferation and migration.

To validate these findings in vivo*,* we established an intracranial xenograft model using U251 cells, given their strong tumorigenicity in nude mice. Luciferase-labeled U251 cells (2 × 10^⁵^) were injected into mice in two groups: knockdown control (sh-NC) and NAT10-knockdown (sh-NAT10). Mice were monitored for body weight every two days and tumor burden every ten days using the IVIS imaging system **(**Fig. 2O**)**. Survival was recorded, and brain tissues were harvested post-mortem. Histological analysis by H&E staining revealed significant tumor size reduction in the sh-NAT10 group beginning at day 10 post-injection, supported by both decreased luciferase signals and tumor mass **(**Fig. 2P–R**)**. Moreover, mice in the sh-NAT10 group experienced slower weight loss from day 20 onward **(**Fig. 2S**)** and had significantly prolonged OS **(**Fig. 2T**)**. Collectively, these data demonstrated that NAT10 played a crucial role in promoting GBM tumor progression and that its disruption can effectively suppress GBM cell growth and invasiveness both in vitro and in vivo.

### Identification of ac4C-modified genes regulated by NAT10

**Fig. 3:**
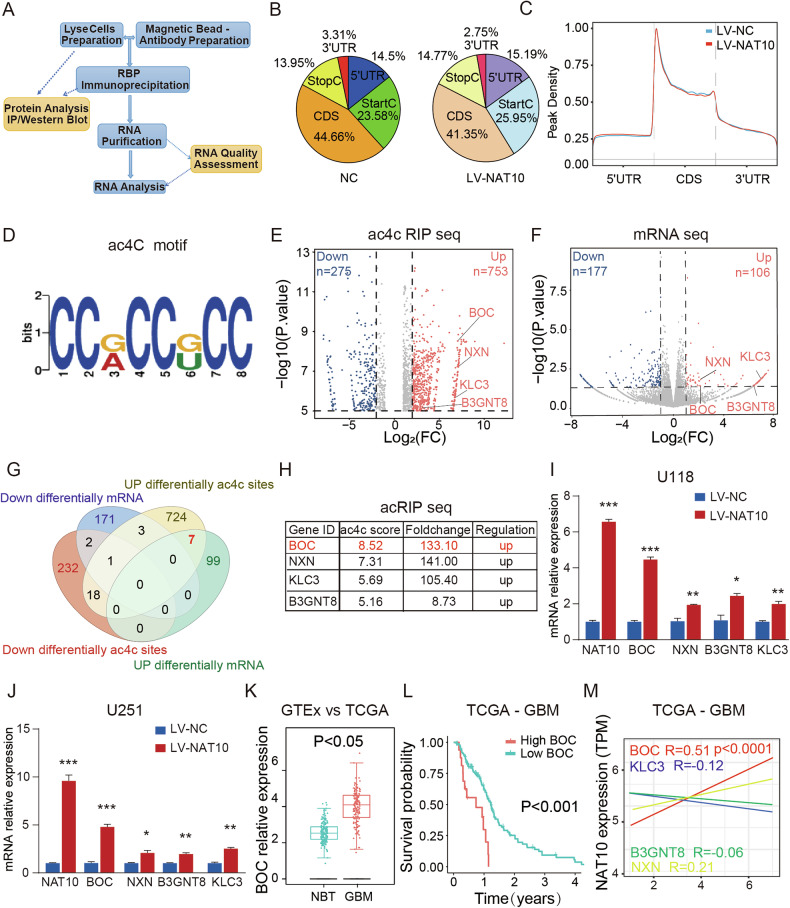
Identification of the profile of ac4C‑modified genes regulated by NAT10. **A** Workflow of RIP procedure. **B**, **C** The location of acetylation modification in ac4c-RIP-seq. **D** The consistent motifs of acetylation modification in ac4C RIP-seq. **E** ac4C RIP sequencing to identify downstream targets of NAT10. **F** The mRNA seq result. **G** A comparison of acetylation gene levels of ac4C-RIP seq and mRNA seq. **H** The analysis of the genes’ acetylation level scores. **I**, **J** The qPCR validation of the four genes RNA level identified in U251 cells. **K** The expression of BOC between NBT (GTEx dataset) and GBMs (TCGA dataset) were downloaded from the GEPIA website. **L** Kaplan‒Meier survival curve of patients with GBM patients in the low and high BOC expression group. **M** Correlation analysis between the top four genes and NAT10 expression. ns *p* > 0.05, *p < 0.05, **p < 0.01, ***p < 0.001.

To elucidate the molecular mechanisms by which NAT10 promoted GBM progression, we performed RNA sequencing (RNA-seq) and ac4C-RNA immunoprecipitation sequencing (acRIP-seq) in U118 LV-NAT10 and control cells. This integrated approach allowed a comprehensive analysis of gene transcriptional profiles alongside mRNA acetylation modifications **(**Fig. 3A**)**. The acRIP-seq data revealed that ac4C peaks were predominantly enriched within coding sequences, particularly in typical CCX motifs **(**Fig. 3B–D**)**. Given NAT10’s function as the sole ac4C writer, we focused on ac4C peaks that increased in abundance upon NAT10 overexpression, identifying 753 hyper-acetylation peaks **(**Fig. 3E**)**. RNA-seq analysis revealed 283 differentially expressed genes, of which 106 were upregulated and 177 downregulated **(**Fig. 3F**)**. The mRNAs exhibiting both hyper-acetylation and upregulation were likely direct substrates of NAT10, including candidates such as BOC, NXN, KLC3, and B3GNT8 **(**Fig. 3G, H**)**.

Notably, BOC, a member of the immunoglobulin superfamily and co-receptor in the hedgehog signaling pathway, displayed the highest ac4C score **(**Fig. 3H**)** [21]. To validate these findings, we performed RT-qPCR to measure the expression of the top four ac4C-scored mRNAs in U118 and U251 cells. All four mRNAs were significantly upregulated following NAT10 overexpression, with BOC showing the most pronounced increase **(**Fig. 3I, J**)**. Bioinformatics analyses demonstrated that BOC mRNA expression was significantly elevated in GBM relative to NBT and was higher in HGGs than in LGGs **(**Figs. 3K, S3A**)**. Kaplan–Meier survival analysis in TCGA cohorts indicated that elevated BOC expression correlated with poorer OS **(**Figs. 3L, S3B**)**. Furthermore, Pearson correlation analysis revealed a positive association between NAT10 and its candidate substrates BOC and NXN in TCGA GBM clinical samples, with the strongest correlation observed for BOC mRNA (R = 0.51, *p* < 0.001) **(**Fig. 3M**)**. Importantly, BOC has previously been identified as an oncogene in GBM and medulloblastoma, promoting tumor development [13]. Our findings suggested that NAT10 catalyzed ac4C modification of BOC mRNA, suggesting BOC as a critical functional target of NAT10 in GBM.

### BOC mRNA as a functionally essential target of NAT10 in GBM

To further confirm that BOC mRNA was a downstream target of NAT10, we analyzed its expression levels. We found that both BOC mRNA and protein levels significantly decreased upon NAT10 knockdown **(**Fig. S3C–E**)**, whereas NAT10 overexpression resulted in a marked increase in BOC expression **(**Fig. S3F–H**)**. Additionally, tumor xenografts derived from GBM cells treated with sh-NAT10 displayed reduced BOC staining **(**Fig. S3I**)**. To elucidate the functional role of BOC in mediating NAT10 activity in GBM, we examined its impact on GBM cell proliferation and migration. BOC was overexpressed using lentivirus and knocked down via shRNA in U251 and U118 cells, confirmed by WB **(**Fig. S4A, B**)**. Consistent with our hypothesis, BOC knockdown inhibited cellular proliferation and migration compared to mock and negative control groups, while BOC overexpression enhanced these processes **(**Fig. S4C–F**)**. To comprehensively validate the functional interplay between NAT10 and BOC in GBM progression, we performed reciprocal rescue experiments. While BOC reconstitution in NAT10-deficient cells significantly restored the impaired proliferative and migratory capacities concomitant with the recovery of BOC protein expression **(**Fig. S5A–D**)**, we further demonstrated that BOC depletion effectively attenuated NAT10-driven proliferation and migration, concurrently with a reduction in BOC levels **(**Fig. S5E–H**)**. These complementary genetic manipulations establish BOC as a critical downstream effector mediating NAT10’s oncogenic functions, confirming an epistatic relationship within the NAT10-ac4C-BOC regulatory axis.

Given that BOC was a target of NAT10-mediated acetylation and served as a critical functional indicator, we hypothesized that BOC might be involved in specific signaling pathways contributing to GBM malignancy. KEGG pathway analysis revealed potential associations between BOC and several key pathways, including Wnt, TGF-β, Hedgehog, and Notch signaling **(**Fig. S6A**)**. Subsequent experimental validation demonstrated that BOC knockdown significantly suppressed the activity of only the Wnt and Hedgehog pathways **(**Fig. S6B**)**. Notably, previous studies have established BOC as a crucial initiator and key component of the Hedgehog signaling pathway [22]. Protein-protein interaction (PPI) network analysis further supported this finding, revealing extensive connections between BOC and Hedgehog pathway molecules **(**Fig. S6C**)**. Additionally, WB experiments confirmed that BOC modulated Hedgehog signaling activity **(**Fig. S6D**)**. Collectively, these results indicated that BOC promoted glioma malignant progression by regulating the Hedgehog signaling pathway.

### **NAT10-mediated ac4C modification influenced BOC mRNA stability and translation**

**Fig. 4:**
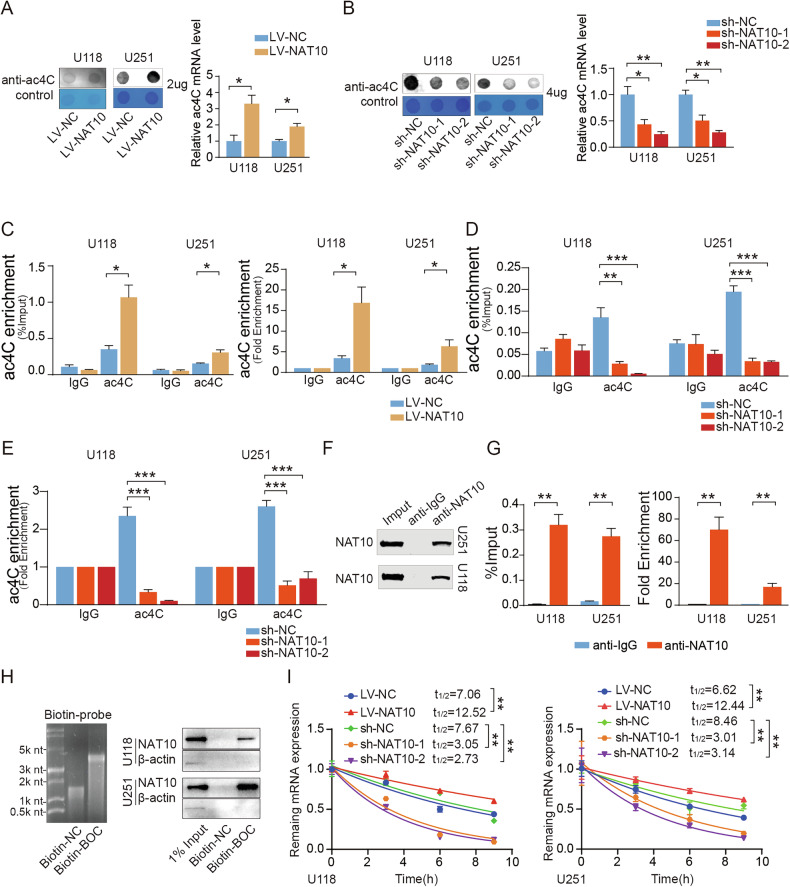
NAT10 interacted with BOC mRNA and regulated BOC mRNA stability in GBM cells. **A**, **B** The total mRNA ac4C level after overexpression and knockdown NAT10 in GBM cells detected by Dot blot assay. **C**–**E** The ac4C-RIP-qPCR assay of BOC mRNA enrichment by ac4C antibody in GBM cells. **F**, **G** NAT10-RIP-qPCR assay of BOC mRNA enrichment by NAT10 antibody in GBM cells. **H** RNA pull-down assay demonstrating the interaction between NAT10 protein and BOC mRNA. Biotin-labeled sense and antisense BOC RNA probes were incubated with cell lysates, and pulled down using streptavidin beads. NAT10 was detected by immunoblotting. **I** The mRNA stability of BOC in NAT10-knockdown and overexpressing stably transfected U251 and U118 cells after treatment with actinomycin D (5 µg/mL). ns *p* > 0.05, *p < 0.05, **p < 0.01, ***p < 0.001.

Consistent with previous findings demonstrating NAT10’s role in regulating ac4C modification in HeLa cells [7], we quantitatively assessed mRNA acetylation patterns in GBM cells using dot blot analysis. Our results revealed a strong positive correlation between NAT10 expression and ac4C mRNA levels, with NAT10 overexpression significantly enhancing mRNA acetylation **(**Fig. 4A**)**. Conversely, NAT10 knockdown resulted in a marked reduction of mRNA ac4C modification **(**Fig. 4B**)**. To specifically assess ac4C modification levels of BOC mRNA, we performed acRIP-seq alongside RT-qPCR, confirming that NAT10 overexpression resulted in increased ac4C modification of BOC mRNA, while NAT10 knockdown had the opposite effect **(**Fig. 4C–E**)**. To elucidate the molecular interaction between NAT10 and BOC mRNA, we first performed RNA immunoprecipitation (RIP) coupled with quantitative PCR (qPCR), which confirmed specific binding of NAT10 protein to BOC mRNA in GBM cells **(**Fig. 4F–G**)**. To further validate this interaction, we conducted in vitro RNA pull-down assays using a synthesized BOC mRNA probe **(**Fig. 4H**)**. Together, these complementary approaches, RIP-qPCR for endogenous detection and pull-down for biochemical verification, demonstrated a direct NAT10-BOC mRNA interaction, providing mechanistic insight into NAT10-mediated ac4C-dependent regulation. Previous studies have suggested that ac4C modifications influences mRNA stability [7]. To explore these properties in BOC mRNA, we assessed its stability via RNA decay assays after actinomycin D treatment. The BOC transcript displayed greater stability in the LV-NAT10 group and significantly reduced stability in the NAT10 knockdown group. Notably, NAT10 overexpression extended the half-life (t_1/2_) of BOC mRNA from 7.06 h to 12.52 h in U118 cells and from 6.62 h to 12.44 h in U251 cells. Conversely, BOC mRNA half-life decreased by over 50% upon NAT10 knockdown **(**Fig. 4I**)**.

**Fig. 5:**
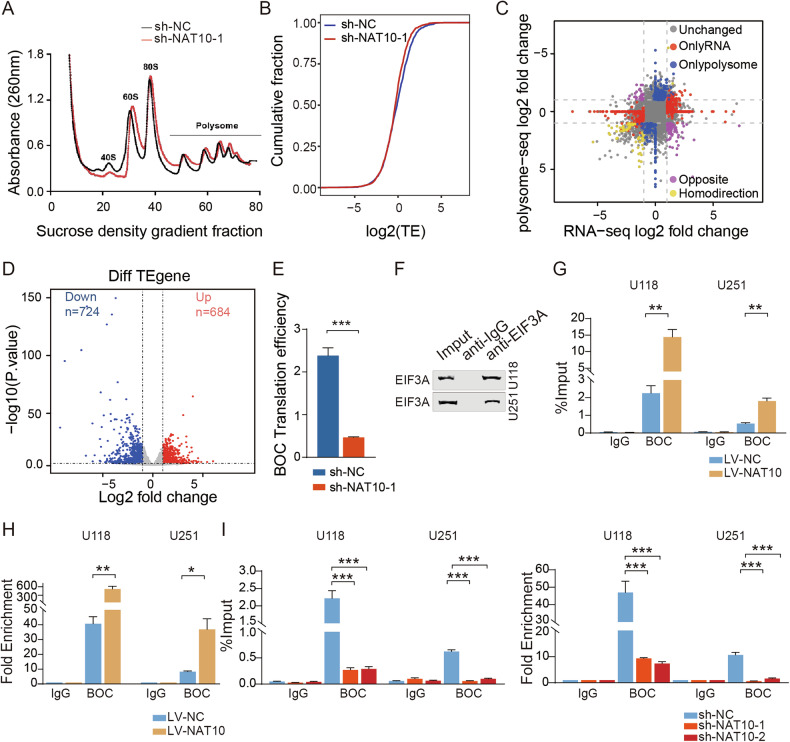
NAT10 regulated BOC mRNA translation in GBM cells. **A** Polysome profile assay showed an overall tendency of translation efficiency after knockdown of NAT10 in U251 cells. **B** Cumulative distribution of translation efficiency showed the treatment group (sh-NAT10) shows a left-shift compared to controls (sh-NC). **C**, **D** Polysome profiling sequencing to identify differential translation efficiency genes. **E** The translation efficiency of BOC in different groups. **F**–**I** EIF3A-RIP analysis of BOC mRNA under different NAT10 expression levels. ns *p* > 0.05, *p < 0.05, **p < 0.01, ***p < 0.001.

Recent studies have established that RNA acetylation, particularly N4-acetylcytidine (ac4C), serves as a critical post-transcriptional modulator of gene expression by influencing mRNA translation efficiency [23]. Our polysome profiling analysis demonstrated that while global translation efficiency remained largely unchanged upon NAT10 depletion **(**Fig. 5A, B), a subset of 1408 transcripts exhibited significant alterations in translational output **(**Fig. 5C, D**)**. Furthermore, ribosome profiling showed that knockdown of NAT10 resulted in a decrease of BOC mRNA in the translating pool **(**Fig. 5E**)**, suggesting that NAT10 also has an impact on the translation of BOC. Mechanistically, given that EIF3A is a core translation initiation factor and a key effector of translational enhancement [24], we investigated its involvement in NAT10-mediated regulation. RIP-qPCR experiments revealed that EIF3A binding to BOC mRNA was strongly dependent on NAT10 activity **(**Fig. 5F–I**)**. These findings collectively demonstrated that NAT10 orchestrated a dual regulatory mechanism, simultaneously stabilizing BOC mRNA through ac4C modification and facilitating its translation via EIF3A recruitment, thereby amplifying oncogenic signaling in GBM.

**Fig. 6:**
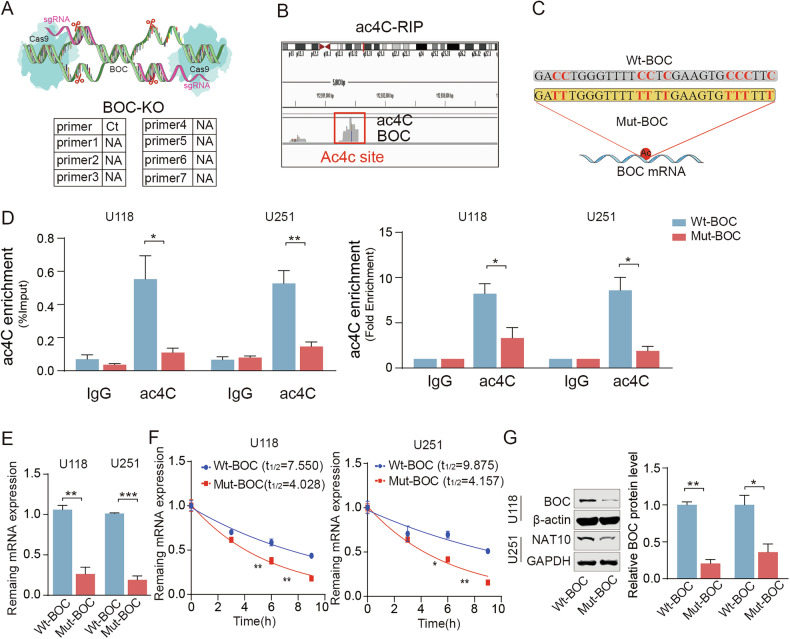
ac4C modification exerted a dual role in regulating both BOC mRNA and protein levels. **A** The schematic diagram of Crispr Cas9 knockout of BOC and Seven pairs of primers were used to detect the outcome of Crispr Cas9 knockout BOC. **B** The binding ac4C site of NAT10 interacted with BOC mRNA. **C** The schematic diagram of BOC mutant plasmid. WT, wild-type vector; Mut, mutant sequence vector of BOC mRNA. **D** Detection of ac4C modification levels on BOC mRNA in cells reconstituted with WT or Mut BOC CDS, as determined by acRIP-qPCR. **E** Endogenous BOC mRNA expression levels in BOC-knockout cells reconstituted with either WT or Mut BOC CDS, measured by RT-qPCR. **F** mRNA stability of BOC in WT and Mut group transfected U251 and U118 cells after treatment with actinomycin D (5 µg/mL). **G** Western blot analysis showed that mutation of the ac4C sites led to a pronounced decrease in BOC protein abundance in BOC-knockout cells reconstituted with WT or Mut BOC CDS. ns *p* > 0.05, *p < 0.05, **p < 0.01, ***p < 0.001.

Furthermore, to directly investigate the functional consequence of ac4C modification on BOC, we utilized CRISPR-Cas9 to knock out BOC and constructed wild-type and mutant BOC-coding sequences (CDS), replacing cytosine (C) with thymine (T) at the ac4C modification sites **(**Fig. 6A–C**)**. Using acRIP, we confirmed that these mutations indeed abolished ac4C modification on BOC mRNA **(**Fig. 6D**)**. In BOC-knockout cells, re-expressing wild-type BOC-CDS or mutant BOC-CDS, results revealed a significant reduction in BOC mRNA levels due to mutations, with half-life for mutant BOC mRNA decreasing from approximately 8–10 h to 4 h **(**Fig. 6E, F**)**, indicating that loss of ac4C modification compromises BOC mRNA stability. Moreover, this post-transcriptional effect was accompanied by a corresponding decrease in BOC protein expression **(**Fig. 6G). Collectively, these results demonstrated that disruption of ac4C modification at specific sites impairs BOC mRNA stability, leading to coordinated downregulation at both transcript and protein levels.

### **Hypoxia-induced NAT10 activation is suppressed by Remodelin in Gliomas**

**Fig. 7:**
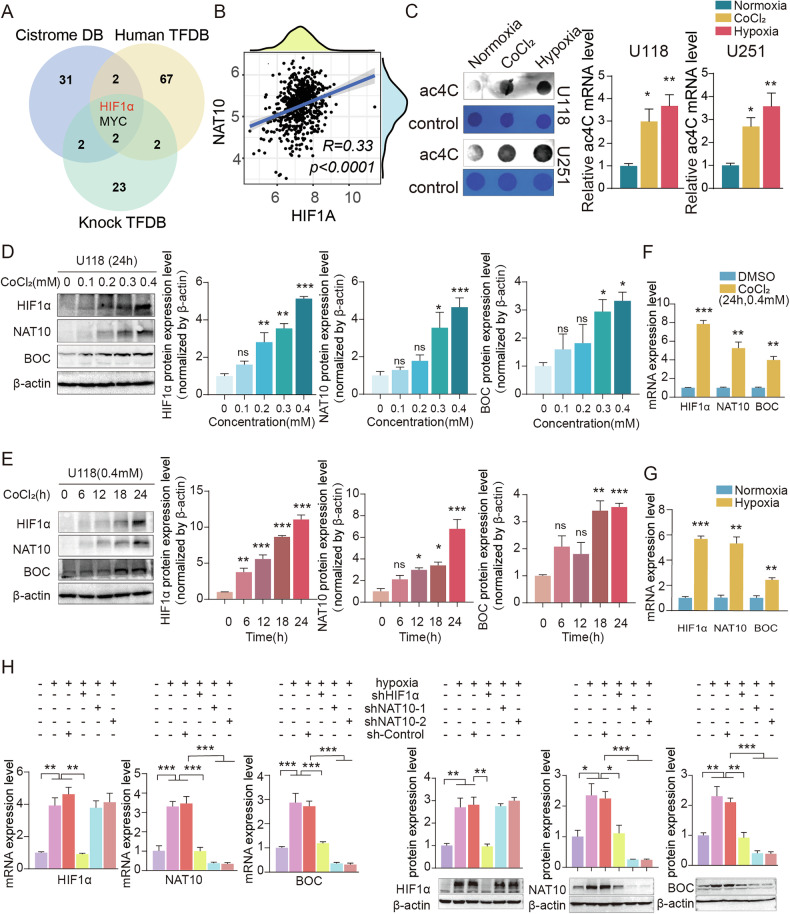
Hypoxia-induced NAT10 activation was suppressed by Remodelin in GBM. **A** The screening process for transcription factor of NAT10 in the Cistrome database, human transcription factor database (TFDB) and knock TFDB. **B** The correlation analysis of HIF1α and NAT10 expression in the TCGA dataset. **C** The mRNA ac4C level in the normoxia and hypoxia conditions was detected by dot blot in GBM cells. **D** The protein expressions of HIF1α, NAT10, and BOC were detected with treating with CoCl_2_ (24 h) for 0, 0.1, 0.2, 0.3, and 0.4 mM in U118 cells. **E** The protein expressions of HIF1α, NAT10, and BOC were detected with treating with CoCl_2_ (0.4 mM) for 0, 6, 12, 18, and 24 h in U118 cells. **F** The mRNA level of HIF1α, NAT10, and BOC were detected by RT-PCR treated by 0.4 mM CoCl_2_ for 24 h in U118 cells. **G** The mRNA level of HIF1α, NAT10, and BOC were detected by RT-PCR treated with normoxia and hypoxia condition in U118 cells. **H** BOC expression in HIF1α-knockdown and NAT10-knockdown stably transfected U118 cells under the normoxia and hypoxia condition. ns *p* > 0.05, *p < 0.05, **p < 0.01, ***p < 0.001.

Hypoxia is a core hallmark of solid tumors and is recognized as a major factor driving the aggressive phenotype of GBM [25]. In this study, we investigated whether hypoxia influenced NAT10 expression and ac4C modification. A recent study reported that NAT10 transcription is regulated by hypoxia-inducible factor 1 alpha (HIF1α) in gastric cancer [26], however, many details remain to be elucidated. Using the Cistrome database, human transcription factor database (TFDB), and knock TFDB database, we identified HIF1α and one additional transcription factor as potential regulators of NAT10 **(**Fig. 7A**)**. Bioinformatics analysis revealed a strong correlation between HIF1α and NAT10 expression in the TCGA-GBM cohort **(**Fig. 7B**)**. To assess the impact of hypoxia on acetylation levels, we performed dot blot assays to measure mRNA acetylation. The results demonstrated that cobalt chloride (CoCl₂) and hypoxic conditions (1% O₂) induced an increase in ac4C in human GBM cells **(**Fig. 7C**)**. As the only known writer of ac4C, NAT10 was upregulated in a dose- and time-dependent manner following CoCl₂ treatment, paralleling the expression patterns of HIF1α and BOC. The peak expression of NAT10 occurred at 24 hours post-CoCl₂ induction, with optimal concentrations of 0.4 mM for U118 cells and 0.3 mM for U251 cells **(**Figs. 7D, E, S7A, B**)**. Similarly, mRNA levels of HIF1α, NAT10, and BOC were significantly increased under both CoCl₂ and hypoxic conditions **(**Fig. 7F, G, S7C, D**)**.

**Fig. 8:**
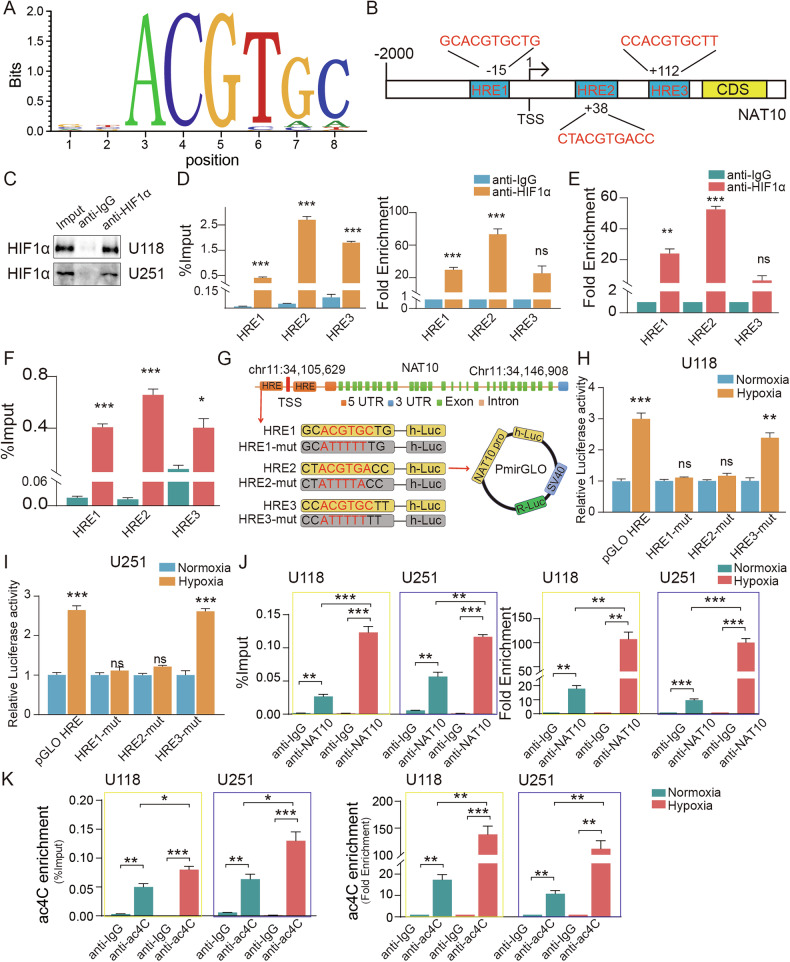
HIF1α directly bound to NAT10 promoter and regulated NAT10 activity in GBM cells. **A** Consistent binding motifs of HIF1α in transcriptional regulation. **B** Design of ChIP-PCR primers of HIF-1α binding regions. **C**–**F** The ChIP results indicated that HRE1/2 on the NAT10 promoter was captured by HIF1α immunoprecipitation in GBM cells. **G** Luciferase reporter Pattern diagram. **H**, **I** Luciferase reporter assays were used to detect relative luciferase activity transiently transfected with a wild type NAT10 promoter linked to luciferase or a HRE mutant NAT10 promoter construct under the normoxia or hypoxia. **J**–**K** acRIP and NAT10 RIP assay of BOC mRNA enrichment by NAT10 and ac4C antibody in GBM cells under hypoxia and normoxia condition. ns *p* > 0.05, *p < 0.05, **p < 0.01, ***p < 0.001.

To elucidate the signaling cascade, we performed knockdown experiments for HIF1α and NAT10 under hypoxic conditions. As expected, HIF1α knockdown diminished both mRNA and protein expression levels of NAT10 and BOC induced by hypoxia. In contrast, NAT10 knockdown only reduced BOC expression without affecting HIF1α levels **(**Figs. 7H, S7E**)**. These findings suggested that hypoxia enhanced the expression of NAT10 and its substrate BOC through HIF1α transcriptional activation of NAT10. Immunofluorescence staining revealed that hypoxia led to the nuclear accumulation of both HIF1α, an effect that was abolished upon HIF1α knockdown **(**Fig. S7F**)**. Bioinformatics analysis using the JASPAR database identified three hypoxia response elements (HREs) within the promoter region of NAT10 **(**Fig. 8A, B**)**. Chromatin immunoprecipitation (ChIP)-qPCR assays demonstrated significant enrichment of HIF1α binding to HRE1 and HRE2 of the promoter region of NAT10, compared to normal IgG control **(**Fig. 8C–F**)**. We subsequently cloned mutant construsts of the HREs into a plasmid luciferase reporter **(**Fig. 8G**)**. The reporter analysis indicated that the construction containing the full-length NAT10 promoter region significantly increased luciferase activity under hypoxic conditions. Among the three mutants, HRE1 and HRE2 mutants resulted in loss of luciferase activity, while HRE3 mutants still maintained luciferase activity under hypoxic conditions. **(**Fig. 8H, I**)**. Finally, RIP followed by RT-qPCR confirmed that hypoxia increased the binding between NAT10 and BOC mRNA, leading to elevated ac4C levels on BOC mRNA **(**Fig. 8J, K**)**. Collectively, our data indicated that the hypoxic tumor microenvironment enhanced NAT10 transcription through HIF1α, subsequently accelerating ac4C modification.

As a well-characterized inhibitor specifically targeting NAT10, Remodelin was investigated for its effects on NAT10 function in glioma cells [27]. Using a range of concentrations and a 24-hour treatment period, we determined the half-maximal inhibitory concentration (IC50) of Remodelin in glioma U251 cells to be 37 μM **(**Fig. S8A**)**. Under the optimized treatment condition (37 μM, 24 h), Remodelin significantly reduced NAT10 protein expression and suppressed its ac4C-mediated mRNA acetylation activity **(**Fig. S8B, C**)**. Importantly, given that hypoxia is a defining characteristic of the tumor microenvironment and may influence drug efficacy, we next investigated whether Remodelin maintains its NAT10-inhibitory capacity under hypoxic conditions. CCK-8 proliferation assays revealed that Remodelin treatment (37 μM, 24 h) under hypoxia (1% O_2_) led to significant growth inhibition of glioma cells **(**Fig. S8D**)**, comparable to its effects under normoxic conditions. Notably, under hypoxic conditions (1% O₂), Remodelin treatment (37 μM, 24 h) significantly reduced ac4C mRNA modification levels, NAT10 and BOC expression level **(**Fig. S8E**)**, demonstrating effective inhibition of NAT10’s acetylation function. These findings demonstrated that Remodelin robustly inhibited NAT10-mediated functions in glioma cells, regardless of oxygen availability, reinforcing its potential as a therapeutic agent targeting NAT10 in glioma treatment.

## Discussion

In addition to genetic factors, epigenomic changes play a crucial role in various cancers and have emerged as an important field for early-stage diagnostics and treatment. ac4C has recently gained attention as a key player in epigenomic research, following the identification of m^6^A methylation, with NAT10 serving as its sole writer. ac4C was first detected on tRNA and rRNA [18, 28, 29] and has recently been recognized as the first acetylation event in mRNA. The oncogenic role of NAT10 and its ability to acetylate mRNA have been demonstrated in bladder, prostate, and gastric cancers [30–32]. Consistent with these findings, our study identifies NAT10 as an oncogene in GBM. Elevated NAT10 expression in GBM patients correlates with poorer prognosis, and its overexpression promotes proliferation and migration in vitro. Notably, in xenograft GBM mouse models, sh-NAT10 effectively impedes GBM progression, suggesting that targeting NAT10 may offer a novel strategy for GBM treatment.

As the only writer of ac4C, NAT10 possesses both acetylation and RNA binding capabilities. Its protein structure includes a conserved region of unknown function (DUF1726), a helicase domain (RecD), a G-N-acetyltransferase domain, and an RNA-binding domain [29]. The RNA-binding domain may determine substrate specificity. Early studies on NAT10 primarily focused on its protein-acetylation activity. For instance, NAT10 acetylates p53 at K120, thereby stabilizing p53 by counteracting Mdm2 activity in colorectal cancer [33]. In breast cancer, NAT10-mediated acetylation of MORC2 regulates cell-cycle checkpoint control and confers resistance to DNA-damaging chemotherapy and radiotherapy [34]. In Daniel Arango’s study [7], the level of ac4C, rather than protein acetylation, was affected by the ablation or re-expression of NAT10 in HeLa and 293 T cells, indicating that RNA is the preferred substrate of full-length NAT10. However, initial investigations into NAT10’s protein-acetyltransferase activity focused on an isoform lacking the N-terminal RNA-interacting region [35, 36]. In our study, we observed changes in ac4C peaks in the GBM transcriptome following NAT10 overexpression, as determined by acRIP-seq. Consistent with previous research, the alterations in ac4C peaks in NAT10-overexpressing cells were primarily located within the coding sequence, particularly in the base sequence CCG.

Our multi-omics approach combining RNA-seq, acRIP-seq, and polysome profiling revealed BOC mRNA as a functional target of NAT10-mediated ac4C modification. Polysome profiling analysis demonstrated that ac4C deposition promotes ribosomal engagement and translational efficiency of BOC mRNA. BOC acts as a co-receptor in the Hedgehog signaling pathway, essential for mediating proper signaling during embryogenesis alongside the canonical receptor PTCH1 and co-receptors GAS1 and CDON [37]. Research regarding BOC in cancer is limited, but a few studies suggest a carcinogenic function; BOC is aberrant expressed in primary breast tumors that develop brain metastases, indicating a role in cell movement [38]. In both human and mouse medulloblastomas, BOC is upregulated and promotes tumor cell proliferation and the expression of proliferative oncogenes at early stages [13]. A recent study further demonstrates that BOC enhances proliferation, migration, and invasion in glioma cells [16], aligning with our functional experiments involving BOC. Our results demonstrate that the knockdown of BOC significantly inhibits GLI1, a key effector of the Hedgehog pathway [39]. Interestingly, while BOC typically functions through the Hedgehog signaling pathway, our findings indicate that, when NAT10 is knocked down (which also reduces BOC, as it is a functional substrate of NAT10), GLI1 activity is markedly upregulated. Similar effects of BOC on Hedgehog signaling have been reported in zebrafish and mouse craniofacial development [15, 40].

The established regulatory paradigm of N4-acetylcytidine (ac4C) modification underscores its potential to enhance both mRNA stability and translational efficiency, acting as a pivotal post-transcriptional mechanism to amplify gene expression [41]. It is important to note, however, that not all acetylated genes are subject to dual regulation at both levels. Our global analyses, integrating RNA-seq and ribosome profiling, reveal that ac4C modification predominantly exerts its effect at the translational level in glioma, as evidenced by the markedly greater number of genes affected in translation (over 200) compared to those altered in mRNA abundance (only 7). Our data reveals that BOC mRNA is modified by ac4C within its coding sequence, with NAT10 directly binding to the BOC transcript. RNA stability analysis indicated a shortened half-life of BOC transcripts after NAT10 knockdown, which was similarly induced by replacing cytosine with thymine in the CDS regions of BOC mRNA. In addition, polysome profiling showed that reduced acetylation levels significantly impair BOC translation efficiency. This was further supported by diminished EIF3A binding on BOC transcripts upon NAT10 knockdown, demonstrating ac4C’s involvement in translation initiation. This is partially consistent with recent research showing that mRNA acetylation regulates translation in a location-specific manner [7]. In this context, our study identifies BOC mRNA as a compelling example of coordinated dual regulation. Specifically, we demonstrate that NAT10-mediated ac4C modification not only enhances the stability of BOC transcripts but also promotes their engagement with the translational apparatus, supported by polysome profiling and reduced EIF3A binding. Together, BOC emerges as a key target wherein the ac4C-NAT10 axis integrates both mRNA stabilization and translational enhancement to fine-tune gene expression in critical cellular processes.

Given the significant effects of hypoxia in the tumor microenvironment, we examined the relationship between ac4C modification and hypoxia. Our findings confirm that hypoxia enhances ac4C deposition in GBM cells. Consistent with prior studies [26], NAT10 transcription is regulated by HIF1α, a key transcription factor in the cellular response to hypoxia. We identified that HIF1α interacts with HRE1 and HRE2 but not HRE3 on the NAT10 promoter, with increased levels of NAT10 induced by CoCl₂ in a dose- and time-dependent manner. Recent reports suggest that NAT10 can also influence HIF1α [26, 42], though not in an ac4C-dependent manner, however according to our data, where NAT10 knockdown did not suppress the elevated levels of HIF1α induced by hypoxia in GBM. While previous research established Remodelin as a NAT10 inhibitor [43], our study provides crucial evidence that this inhibition remains effective even in hypoxic conditions. Most importantly, our pharmacological studies with Remodelin demonstrate this pathway remains targetable in the challenging hypoxic tumor microenvironment. Treatment with Remodelin (37 μM, 24 h) under 1% O₂ conditions effectively suppressed NAT10 activity, as evidenced by reduced ac4C modification of BOC mRNA and impaired tumor cell proliferation.

In conclusion, our studies highlight the critical role of NAT10 in GBM pathogenesis, characterized by its promotion of proliferation and migration, and uncover a previously unrecognized ac4C target, BOC mRNA, in GBM cells. Furthermore, our work indicates that HIF1α influences NAT10 and its ac4C writer function through transcriptional activation. Most importantly, we demonstrate that Remodelin effectively inhibits NAT10’s oncogenic functions even under hypoxic conditions, underscoring its therapeutic potential. Thus, NAT10 emerges as a promising biomarker and potential therapeutic target for GBM.

## Materials and methods

### **Clinical specimens**

The commercial GBM tissue microarray was obtained from Bioaitech Company (N783701, China). All GBM tissues and cancer-adjacent tissues (CATs) used in this study were sourced from the Neurosurgery Department of the Second Affiliated Hospital of Nanchang University (NCUSAH). This study received approval from the Medical Ethics Committee of NCUSAH and was conducted in accordance with the approved guidelines. Informed consent was obtained from all human participants involved in this study prior to data collection. The gene expression profiling was obtained from the Cancer Genome Atlas (TCGA; https://portal.gdc.cancer.gov/), Chinese Glioma Genome Atlas (CGGA; www.cgga.org.cn/) and Gene Expression Omnibus (GEO; www.ncbi. nlm. nih.gov/geo/) databases and Sanger-box platform (http://sangerbox.com/home.html#). The inclusion criteria for glioma were as follows: (1) patients diagnosed with glioma with OS of more than 30 days, (2) patients with expression data, and (3) patients with primary glioma. The clinical characteristics of the patients are summarized in Table 1.

**Table 1:** The imformation of patients with GBM.

Features	TCGA (*n* = 629)	CGGA (*n* = 309)	GSE108474 (*n* = 305)	NCUSAH (*n* = 30)
**Overall survival (years)**				
Median (range)	1.38 (0.09–17.60)	1.99 (0.09–13.18)	0.72 (0.08–8.28)	0.96 (0.21–2.03)
<5	573 (91.10%)	212 (68.61%)	302 (99.02%)	30 (100.00%)
>=5	56 (8.90%)	97 (31.39%)	3 (0.98%)	0 (0.00%)
**Age**				
Median (range)	46 (14–87)	42 (8–79)	50 (1–85)	58 (39–76)
<median age	307(48.80%)	139 (44.98%)	142 (46.56%)	14 (46.67%)
>=median age	322 (51.20%)	170 (55.02%)	163 (53.44%)	16 (53.33%)
NA	0 (0.00%)	0 (0.00%)	0 (0.00%)	0 (0.00%)
**Gender**				
Male	359 (57.07%)	194 (62.78%)	163 (53.44%)	18 (60.00%)
Female	270 (42.93%)	115 (37.22%)	102 (33.44%)	12 (40.00%)
NA	0 (0.00%)	0 (0.00%)	40 (13.12%)	0 (0.00%)
**WHO grade**				
WHO II	231 (36.72%)	97 (31.40%)	76 (24.92%)	/
WHO III	246 (39.11%)	73 (23.62%)	65 (21.31%)	/
WHO IV	152 (24.17%)	135 (43.69%)	164 (53.77%)	30 (100.00%)
NA	0 (0.00%)	4 (1.29%)	0 (0.00%)	0 (0.00%)
**IDH mutation status**				
Mutant	400 (63.59%)	165 (53.40%)	/	/
Wild	220 (34.98%)	143 (46.28%)	/	/
NA	9 (1.43%)	1 (0.32%)	/	/
**1p/19q codeletion status**				
Non-codeletion	468 (74.40%)	239 (77.35%)	/	/
Codeletion	156 (24.80%)	62 (20.06%)	/	/
NA	5 (0.80%)	8 (2.59%)	/	/
**MGMT promoter status**				
Methylated	448 (71.22%)	151 (48.87%)	/	/
Unmethylated	149 (23.69%)	140 (45.31%)	/	/
NA	32 (5.09%)	18 (5.82%)	/	/

### Cell culture

The U87, T98, U118, and U251 cell lines were obtained from the American Type Culture Collection (ATCC) in the United States, while the normal human astrocytes (NHA) were purchased from Puno Sai Life Technology Co., Ltd. (Wuhan, China). All cell lines, except U87, were cultured in Dulbecco’s Modified Eagle’s Medium/F12 (DMEM) supplemented with 10% fetal bovine serum (Excell Bio). U87 cells were cultured in Minimum Essential Medium (MEM) with 10% fetal bovine serum (Excell Bio). All cell lines were maintained in an incubator at 37°C with 5% CO2. For hypoxia treatments, cells were plated at the desired density before placement in a hypoxia chamber (MCO-18M; PHCbi Technologies) maintained at 0.1% oxygen for differents time.

### Plasmid construction and transfection

The OE-NAT10 plasmid and negative control plasmid were commercially obtained from SWS Technology Co., Ltd. (Tianjin, China). The myc-T7-BOC plasmid was constructed from the pCDH-puro-myr-HA-Akt1 vector (Addgene; Cat# 46969) using the Clon Express II One Step Cloning Kit (Vazyme; Cat# C112). The mutant myc-T7-BOC, pGLO-NAT10-WT-Luc, and pGLO-NAT10-Mut-Luc plasmid was generated from the wild-type myc-T7-BOC and OE-NAT10 plasmid using the Mut Express II Fast Mutagenesis Kit V2 (Vazyme; Cat# C214). All cloned plasmids were verified by DNA sequencing. Lipofectamine 3000 (Invitrogen, USA) was used for the transient transfection of these plasmids into cells.

### Lentiviral construction and transfection

The sh-HIF1α, sh-NAT10 lentiviral, sh-BOC lentiviral, CRISPR-Cas9 lentiviral, and sgRNA lentiviral constructs were synthesized by OBiO Technology Co., Ltd. (Shanghai, China). The infectious lentiviruses were introduced into cells following the manufacturer’s instructions from OBiO Technology. The construction process for the OE-NAT10 and OE-BOC lentivirals was as follows: 293 T cells were seeded in six-well plates. Two hours prior to transfection, the cells were replenished with fresh DMEM. The transfection mixture consisted of 2 μg of the target plasmid (OE-NAT10 plasmid/myc-T7-BOC plasmid), 1.5 μg of the PSPAX2 packaging plasmid, 1 μg of the PMD membrane plasmid, and 13.5 μL of EZ-Transfection reagent (Life-iLab; Cat# AC04L091) in 250 μL of Opti-MEM. After mixing, the solution was incubated for 15 minutes before being added to the 293 T cells. Six hours later, 4 mL of fresh DMEM was added to each well. For viral collection, the supernatant was harvested 24 hours post-transfection and stored at 4 °C. This supernatant was then supplemented with fresh DMEM. After another 24 hours, the supernatant was collected again into 50 mL centrifuge tubes. Centrifugation was performed at 4 °C at 2000 × *g* for 10 minutes. The supernatant was filtered through a 0.45 μm filter and transferred to new centrifuge tubes, then stored at −80 °C for subsequent use in infecting glioma cells. The p shRNA information used in the study in the Table 2.

**Table 2:** The primer and shRNA information used in the study.

primers	sequence
RPL30 F	AAAAAGTCGCTGGAGTCGAT
RPL30 R	AAAGCTGGGCAGTTGTTAGCGAGA
OE-BOC F	acctccatagaagattctagaATGCTGCGTGGGACGATG
OE-BOC R	cggatccgatttaaattcgaaCTAAATTGTGAGAGGTGGTGTTTCA
Mut-OE-BOC F	ATACGACTCACTATAGGGATGCTGCGTGGGACGATG
Mut-OE-BOC R	CCCTATAGTGAGTCGTATTATCTAGAATCTTCTATGGAGGTCAAAACA
ac4C BOC F	CGTCACCTTCATCCCCTTCTGC
ac4C BOC R	GTCCACTGATGCCACTGAGGT
ACTIN-F	TGGAACGGTGAAGGTGACAG
ACTIN-R	CGCATCTCATATTTGGAATGACT
NAT10 F	CTGAGAATAAGACCACGACGACA
NAT10 R	GCAATCCAGGCACAGCAAGT
BOC F	GCACCTCCAAGACAGACTCATA
BOC R	AGAGATGGTCCAATCGTCAGAG
HIF1α F	CTGAGGGGACAGGAGGATCA
HIF1α R	AAAGGCAAGTCCAGAGGTGG
CHIP-HRE1 F	TCCGCTTCTGGTGGCTTACG
CHIP-HRE1 R	TCAGGAACTGGTAGACAGCAC
CHIP-HRE2 F	TCTACCAGTTCCTGAGAGGGA
CHIP-HRE2 R	TCCGAAAGAGAAGGCACAGC
CHIP-HRE3 F	GCTGTGCCTTCTCTTTCGGA
CHIP-HRE3 R	GACACCGATCTTGGCATCCA
NXN F	GTGATCCGAGATGACCCAGAA
NXN R	GCGGAGAAATAGACGCCCAC
KLC3 F	GCCACGCTCAACAACTTGG
KLC3 R	CGCTCCACGTCCTCAAACTT
B3GNT8 F	GTCCCATTCAACCAGACGCTC
B3GNT8 R	GGGCACATAGAAGGGTCCTC
VF-BOC-1 F	CCAGGACTTCAAGTTAGATGTGC
VF-BOC-1 R	CCACTCTTGTTTGACGCTGT
VF-BOC-2 F	ACCCAGTGACCCAGGAAGTGA
VF-BOC-2 R	TGGTGGTGTCGATGAGGAGGT
VF-BOC-3 F	TGCTGTGGCTGAGGAATGCTG
VF-BOC-3 R	GTTGCCGAGTTTGGAGGGTGA
VF-BOC-4 F	GCCAATCCTCTACTATGTGGTGAAA
VF-BOC-4 R	GGGTCAAGTCTGGTGAGGGTCA
VF-BOC-5 F	ACCTCCTACAAGTTTCGAGTCCG
VF-BOC-5 R	CCGCATCCGTGAAGGTGATAT
VF-BOC-6 F	GCGACCTGCCCTATCTGATTGTCGG
VF-BOC-6 R	TGCCACTGAGGTAGGGCTGTC
VF-BOC-7 F	CCAGTCAGGGGTGAGGAGA
VF-BOC-7 R	CTCACTTGGCAGGAGTCAGG
sgRNABOC-1	GAGCCTCCCATTACCGTGCG
sgRNABOC-2	GAACGAGATGCGGCGCCCCT
sgRNABOC-3	CCGGGAGCGACGCAGCTTGA
sgRNABOC-4	ACCCTCCTGCCCGTATACTA
sgRNABOC-5	CCCAAGTCTAGCCCGGACGA
sgRNABOC-6	CGTAGGGGCCTACGTAGGAC
Mut HRE1 F	GCGCTTgcgcattttttgTCTACCAGTTCCTGAGAGGGACG
Mut HRE1 R	caaaaaatgcgcAAGCGCATTAAAGTCGCAGAG
Mut HRE2 F	ctattttaccCGGACACCAGGCATACGCTAGGG
Mut HRE2 R	GGTGTCCGggtaaaatagTAAGCCTGGCTCCGCGGC
Mut HRE3 F	GCtcccatttttttCCCCTTCTCCACTGGCTGG
Mut HRE3 R	GGGGaaaaaaatgggaGCACGGAACAACTCCGAAAG
Mut BOC F	tgggttttttttgaagtgttttttCACCCTCCTGCCCGTATACTAT
Mut BOC R	cacttcaaaaaaaacccaaatctattatatattcTTTGCTTAGACCAGGCCCTCC
clone-BOC F	ggagacccaagcttcccaccaccatgggccaccATGCTGCGTGGGACGATG
clone-BOC R	atccgatttaaattcgaattcCTAAATTGTGAGAGGTGGTGTTTCA
GLI1 For	AGCGTGAGCCTGAATCTGTG
GLI1 Rev	CAGCATGTACTGGGCTTTGAA
SMAD2 For	CGTCCATCTTGCCATTCACG
SMAD2 Rev	CTCAAGCTCATCTAATCGTCCTG
SMAD3 For	CCATCTCCTACTACGAGCTGAA
SMAD3 Rev	CACTGCTGCATTCCTGTTGAC
β-Catenin For	CATCTACACAGTTTGATGCTGCT
β-Catenin Rev	GCAGTTTTGTCAGTTCAGGGA
Notch1 For	GAGGCGTGGCAGACTATGC
Notch1 Rev	CTTGTACTCCGTCAGCGTGA
JAG1 For	GTCCATGCAGAACGTGAACG
JAG1 Rev	GCGGGACTGATACTCCTTGA
RBPJ For	CGGCCTCCACCTAAACGAC
RBPJ Rev	TCCATCCACTGCCCATAAGAT
HES1 For	TCAACACGACACCGGATAAAC
HES1 Rev	GCCGCGAGCTATCTTTCTTCA

shRNA	sequence
**NAT10-1 stealth RNAi:**	**GCAATTGTACACAGTGACTAT**
**NAT10-2 stealth RNAi:**	**GAGCAUGGACCUCUCUGAAUACAUA**
**BOC-1 stealth RNAi:**	**ACCTCCAAGACAGACTCATAT**
**BOC-2 stealth RNAi:**	**CCCGTATACTATGGTGCCATT**
**HIF1a stealth RNAi:**	**GGGATTAACTCAGTTTGAACT**
**control RNAi**	**CCTAAGGTTAAGTCGCCCTCG**

### RNA isolation and Quantitative real-time PCR

Total RNA was extracted using the Simply P Kit (Bioer; Cat# BSC52S1) and subsequently reverse transcribed with the HiScript II 1st Strand cDNA Synthesis Kit (Vazyme; Cat# R211-01). Quantitative real-time PCR (qPCR) was performed using the 2X M5 HiPer Realtime PCR Supermix (Mei5 Biotech; Cat# MF013-01) on a qTOWER3 series fluorescence quantitative gene amplification instrument (Jena; Germany). The primer information used in the study in Table 2.

### Western blot assay

Cell and patient tissue lysates were obtained using a radioimmunoprecipitation assay buffer containing protease inhibitors (Solarbio; Cat# R0010). For Western blot analysis, the following antibodies were used: NAT10 (Proteintech; Cat# 67465-1-Ig, Cat# 13365-1-AP), HIF1α (Proteintech; Cat#20960-1-AP), BOC (ABclone; Cat#A7174, Immunoway; Cat#YN2231), E-Cadherin (Proteintech; Cat#20874-1-AP), N-Cadherin (Proteintech; Cat#22018-1-AP), Vimentin (Proteintech; Cat#10366-1-AP), PCNA (Proteintech; Cat# 10205-2-AP), GLI1 (Proteintech; Cat#66905-1-Ig), GAPDH (Proteintech; Cat#10494-1-AP) and Beta ACTIN (Proteintech; Cat#66009-1-Ig). Total protein was extracted using RIPA buffer (Solarbio; Cat#R0010), and protein concentrations were determined using the BCA Protein Assay Kit (Beyotime; Cat#P0010). The total protein lysates were separated by 10% SDS-PAGE gel electrophoresis and subsequently transferred to PVDF membranes (Millipore; Cat# YZ-ISEQ00010). The PVDF membranes were incubated with primary antibodies, and an automatic chemiluminescence image analysis system (Tanon, Shanghai) was used to visualize specific protein bands.

### ChIP-qPCR assays

The Simple CHIP Plus Sonication Chromatin IP Kit (Cell Signaling Technology; Cat#56383) was employed for the Chip Immunoprecipitation (CHIP) experiment. Essentially, the cell lysate is ultrasonically fragmented into 200–1000 nt sized fragments of DNA after 37% formaldehyde cross-linking and fixation management of the cell, and 1 μg HIF1α (Proteintech; Cat#20960-1-AP), IgG (Proteintech; Cat#30000-0-AP) or positive antibodies and 30 µL Protein G magnetic beads were effectively mixed and interacted with diluted cell lysates. Fragmented DNA was obtained after protein and RNA digestion. Gene expression was adjusted to input thereafter qPCR. Indeed, the ChIP-PCR product underwent further validation through 1% agarose gel electrophoresis.

### Luciferase Reporter Gene Assay

The promoter activity of NAT10 in GBM cells was measured by luciferase assay (UElandy; Cat#F6075S). Briefly, GBM cells were treated with 1%O_2_ and transfected with pGLO-NAT10-WT-Luc, or pGLO-NAT10-Mut-Luc containing the NAT10 promoter for 24 h.

### Immunofluorescence assay

The cells were placed in a six-well plate with cell slides, their density was increased to 70%, followed by PBS washing three times for 5 minutes each, fixation in 4% paraformaldehyde at room temperature (RT) for 15 minutes, 0.5% TrixonX-100 RT penetration for 20 minutes and blocking with 10% goat serum at 37 °C for 1 hour. Subsequently, the mixture was incubated with anti-HIF1α (Proteintech; Cat#66730-1-Ig; 1:200) and anti-NAT10 (Proteintech; Cat#13365-1-AP; 1:200) antibodies in 5% goat serum overnight at 4 °C. Additionally, the mixture was also incubated at RT with the second antibodies conjugated with Goat Anti-Rabbit IgG H&L/AF555 (Bioss; Cat#bs-0295G-AF555; 1:500) or Goat Anti-Mouse IgG H&L/AF594 (Bioss; Cat#bs-0296G-AF594; 1:500) for 2 hours. Finally, the cells were stained with DAPI for 40 seconds and imaged using a confocal microscope (Leica; Germany). The GBM cells were mixed with primary antibody overnight at 4 °C followed by multicolor immunofluorescence staining using the Treble-Fluorescence Immunohistochemistry Rabbit Kit according to the manufacturer’s protocol (Immunoway; Cat# RS0035).

### Immunochemistry assays

This study utilized a commercial GBM tissue microarray (Bioaitech; Cat. No. N783701) for analysis, with the detailed information provided in Table 3. The clinical sections were reviewed by two neuropathologists. Surgical tissues and GBM xenografts were fixed in 4% paraformaldehyde (PFA) and sectioned into 3-μm slices. The sections were deparaffinized, rehydrated, and treated with 3% hydrogen peroxide. Antigen retrieval was performed by boiling in 0.01 M sodium citrate buffer (pH 6.0, Cat No. G1202, Servicebio, Wuhan, China). Following blocking with 5% bovine serum albumin (BSA), the slides were incubated with NAT10 and BOC antibodies overnight at 4°C, and then with secondary antibodies at 37°C for 2 hours. Detection was carried out using DAB staining, followed by hematoxylin counterstaining. Images were captured using a Leica microscope (Wetzlar, Germany) and quantified with ImageJ software using the “IHC Profiler” plugin [44]. The H-score was calculated as: H-score = 0* Percentage contribution of Negative + 1* Percentage contribution of Low positive + 2* Percentage contribution of positive + 3* Percentage contribution of High positive.

**Table 3:** The commercial GBM tissue microarray N783701 information.

N783701 Specs								
Microchip code:	N783701							
Microchip name:	Brain glioblastoma (Grade 1-4) tissue array with normal brain tissue							
Microchip instruction:	Brain glioblastoma (Grade 1-4) with normal brain tissue array, containing 32 glioblastoma, 5 adjacent normal cerebrum tissue and 2 cerebrum tissue, duplicate cores per case							
Sampling method:	Duplicate cores per case							
Points:	78							
Cases:	34							
Sample diameter:	1.5mm							
Detailed information of tissue array panel								
No.	Age	Sex	Organ	Pathology diagnosis	Grade	Type	Clinical diagnosis	Primary/Metastatic
A1	41	F	Brain	Astrocytoma	1	Malignant	Brain tumor	Primary
A2	41	F	Brain	Astrocytoma	1-2	Malignant	Brain tumor	Primary
A3	44	F	Brain	Astrocytoma	1-2	Malignant	Brain tumor	Primary
A4	44	F	Brain	Astrocytoma	1-2	Malignant	Brain tumor	Primary
A5	62	M	Brain	Astrocytoma	1-2	Malignant	Brain tumor	Primary
A6	62	M	Brain	Astrocytoma	1-2	Malignant	Brain tumor	Primary
A7	42	M	Brain	Astrocytoma	1-2	Malignant	Brain tumor	Primary
A8	42	M	Brain	Astrocytoma	1-2	Malignant	Brain tumor	Primary
A9	46	M	Brain	Astrocytoma	1-2	Malignant	Brain tumor	Primary
A10	46	M	Brain	Astrocytoma	1-2	Malignant	Brain tumor	Primary
B1	43	F	Brain	Astrocytoma	1-2	Malignant	Brain tumor	Primary
B2	43	F	Brain	Astrocytoma	1-2	Malignant	Brain tumor	Primary
B3	33	F	Brain	Astrocytoma	1-2	Malignant	Brain tumor	Primary
B4	33	F	Brain	Astrocytoma	1-2	Malignant	Brain tumor	Primary
B5	46	M	Brain	Astrocytoma	1-2	Malignant	Brain tumor	Primary
B6	46	M	Brain	Astrocytoma	1-2	Malignant	Brain tumor	Primary
B7	49	M	Brain	Astrocytoma	2	Malignant	Brain tumor	Primary
B8	49	M	Brain	Astrocytoma	2	Malignant	Brain tumor	Primary
B9	50	M	Brain	Astrocytoma	2	Malignant	Brain tumor	Primary
B10	50	M	Brain	Astrocytoma	2	Malignant	Brain tumor	Primary
C1	38	M	Brain	Astrocytoma	2	Malignant	Brain tumor	Primary
C2	38	M	Brain	Astrocytoma	2	Malignant	Brain tumor	Primary
C3	57	M	Brain	Astrocytoma	2	Malignant	Brain tumor	Primary
C4	57	M	Brain	Astrocytoma	2	Malignant	Brain tumor	Primary
C5	44	F	Brain	Astrocytoma	2-3	Malignant	Brain tumor	Primary
C6	44	F	Brain	Astrocytoma	2-3	Malignant	Brain tumor	Primary
C7	44	M	Brain	Astrocytoma	2-3	Malignant	Brain tumor	Primary
C8	44	M	Brain	Astrocytoma	2-3	Malignant	Brain tumor	Primary
C9	66	M	Brain	Astrocytoma	2-3	Malignant	Brain tumor	Primary
C10	66	M	Brain	Astrocytoma	2-3	Malignant	Brain tumor	Primary
D1	20	M	Brain	Anaplastic astrocytoma	3	Malignant	Brain tumor	Primary
D2	20	M	Brain	Anaplastic astrocytoma	3	Malignant	Brain tumor	Primary
D3	23	M	Brain	Anaplastic astrocytoma	3	Malignant	Brain tumor	Primary
D4	23	M	Brain	Anaplastic astrocytoma	3	Malignant	Brain tumor	Primary
D5	39	F	Brain	Anaplastic astrocytoma	3	Malignant	Brain tumor	Primary
D6	39	F	Brain	Anaplastic astrocytoma	3	Malignant	Brain tumor	Primary
D7	47	M	Brain	Anaplastic astrocytoma	3	Malignant	Brain tumor	Primary
D8	47	M	Brain	Anaplastic astrocytoma	3	Malignant	Brain tumor	Primary
D9	37	F	Brain	Anaplastic astrocytoma	3	Malignant	Brain tumor	Primary
D10	37	F	Brain	Anaplastic astrocytoma	3	Malignant	Brain tumor	Primary
E1	48	M	Brain	Anaplastic astrocytoma	3	Malignant	Brain tumor	Primary
E2	48	M	Brain	Anaplastic astrocytoma	3	Malignant	Brain tumor	Primary
E3	59	F	Brain	Anaplastic astrocytoma	3	Malignant	Brain tumor	Primary
E4	59	F	Brain	Anaplastic astrocytoma	3	Malignant	Brain tumor	Primary
E5	68	F	Brain	Anaplastic astrocytoma	3	Malignant	Brain tumor	Primary
E6	68	F	Brain	Anaplastic astrocytoma	3	Malignant	Brain tumor	Primary
E7	49	M	Brain	Anaplastic astrocytoma	3-4	Malignant	Brain tumor	Primary
E8	49	M	Brain	Anaplastic astrocytoma	3-4	Malignant	Brain tumor	Primary
E9	36	F	Brain	Anaplastic astrocytoma	3-4	Malignant	Brain tumor	Primary
E10	36	F	Brain	Anaplastic astrocytoma	3-4	Malignant	Brain tumor	Primary
F1	33	F	Brain	Anaplastic astrocytoma	3-4	Malignant	Brain tumor	Primary
F2	33	F	Brain	Anaplastic astrocytoma	3-4	Malignant	Brain tumor	Primary
F3	40	M	Brain	Glioblastoma	4	Malignant	Brain tumor	Primary
F4	40	M	Brain	Glioblastoma	4	Malignant	Brain tumor	Primary
F5	45	M	Brain	Glioblastoma	4	Malignant	Brain tumor	Primary
F6	45	M	Brain	Glioblastoma	4	Malignant	Brain tumor	Primary
F7	37	M	Brain	Glioblastoma	4	Malignant	Brain tumor	Primary
F8	37	M	Brain	Glioblastoma	4	Malignant	Brain tumor	Primary
F9	25	M	Brain	Glioblastoma	4	Malignant	Brain tumor	Primary
F10	25	M	Brain	Glioblastoma	4	Malignant	Brain tumor	Primary
G1	42	F	Brain	Glioblastoma	4	Malignant	Brain tumor	Primary
G2	42	F	Brain	Glioblastoma	4	Malignant	Brain tumor	Primary
G3	13	F	Brain	Glioblastoma	4	Malignant	Brain tumor	Primary
G4	13	F	Brain	Glioblastoma	4	Malignant	Brain tumor	Primary
G5	62	M	Brain	Gliosis	-	Adjacent	-	-
G6	62	M	Brain	Gliosis	-	Adjacent	-	-
G7	44	F	Brain	Adjacent normal cerebrum tissue	-	Adjacent	-	-
G8	44	F	Brain	Adjacent normal cerebrum tissue	-	Adjacent	-	-
G9	42	M	Brain	Adjacent normal cerebrum tissue	-	Adjacent	-	-
G10	42	M	Brain	Adjacent normal cerebrum tissue	-	Adjacent	-	-
H1	43	F	Brain	Adjacent normal cerebrum tissue	-	Adjacent	-	-
H2	43	F	Brain	Adjacent normal cerebrum tissue	-	Adjacent	-	-
H3	20	M	Brain	Adjacent normal cerebrum tissue	-	Adjacent	-	-
H4	20	M	Brain	Adjacent normal cerebrum tissue	-	Adjacent	-	-
H5	55	F	Brain	Cerebrum tissue	-	Normal	-	-
H6	55	F	Brain	Cerebrum tissue	-	Normal	-	-
H7	35	M	Brain	Cerebrum tissue	-	Normal	-	-
H8	35	M	Brain	Cerebrum tissue	-	Normal	-	-

### Transwell migration assay

Migration analysis was conducted using a Corning transwell chamber equipped with a membrane featuring an 8 µm chamber (Corning; Cat#3524). Subsequently, Cells were planked into the upper chamber that contained the serum-free medium. In the lower chamber, the medium was supplemented with a medium supplemented with 10% fetal bovine serum and 1% antibiotic/antimycotic. After 24 h of incubation, non-migration cells on the surface of the chambers were gently wiped away. The migration cells were fixed with 4% paraformaldehyde (Solarbio; Cat#P1110) for 30 minutes and then stained with 1% Crystal Violet Ammonium (Solarbio; Cat# G1062). Images of the migration cells were obtained via a Leica Microsystems CMS GmbH microscope. Each experiment was performed in duplicate to ensure the reproducibility and reliability of the results.

### Wound healing assay

Approximately 5 × 10^5^ cells were grown in a medium with 2% fetal bovine serum on 6-well plates. Using a 1,000 L pipette tip, streaks were formed across the monolayer once the cells had attained 90–100% confluence. Subsequently, PBS was used to wash the cells twice. To evaluate cell migration, cells were further cultured in 2% fetal bovine serum medium at 37 °C and 5% CO_2_ for 24 hours to minimize the impact of cell proliferation. By a Leica microscope (Leica, Germany), images of the cells were obtained at 0, 24, and 48 hours after the wounds were created.

### CCK8 assay

Glioma cells were seeded into 96-well plates at a density of 3,000 cells per well. At specified time points (0, 24, 48, and 72 hours), 10 μL of CCK-8 solution was added to each well and incubated for 2 hours. The absorbance was measured at 450 nm using a microplate reader (Bio-Rad, USA). Each experiment was performed in triplicate.

### ac4C RIP-seq and RNA-seq

After an overexpression intervention was implemented on NAT10, ac4C RIP-seq, and RNA-seq were obtained, which was provided by Shanghai Cloud Series Biotech Co., Ltd. The process is as follows: RNA was randomly fragmented into approximately 200 nt. Protein A/G magnets and ac4C antibodies were incubated at RT for 1 hour to bind the antibody to the magnets. The RNA fragments were then incubated for 4 hours with the complex in rotation at 4 °C to bind the RNA to the antibody. The RNA/antibody complex was washed off from the magnetic bead with protease K, and the RNA was extracted with phenol: chlorine. An RNA sequencing library was built using the NEB Next® Ultra II Directional RNA Library Prep Kit reactor box. The library was quality-controlled with the Agilent 2100 biological analyzer, and then high-flow sequencing was carried out on the Nova-Seq sequencers of Illumina. The ac4C score = -log10 (P-value). The results of ac4C RIP-seq combined RNA-seq were shown in Supplementary Table 1.

### NAT10/ac4C RNA immunoprecipitation

The RNA immunoprecipitation (RIP) experiment was carried out by the Magna RIP Kit protocol (Millipore; Cat#17-701). Briefly, 5 μg NAT10, ac4C, IgG or positive antibodies, and 50 μL magnetic beads were properly combined and infused with lysates. Following the digestion of proteins, RNAs were collected. Subsequently, RT-qPCR was carried out, and gene expression was adjusted to the input. Each RIP fraction was normalized to the input to account for RNA sample differences: ΔCt[normalized RIP] = (Ct[RIP]) − (Ct[input] – log2 (input dilution factor)), where Input Dilution Factor = (fraction of the input chromatin saved)-1. %Input for each RIP fraction was calculated as follows: %input = 2^ ( − ΔCt[normalized RIP]) ×100%. Enrichment was calculated relative to background rabbit IgG antibody, ΔCt[normalized RIP] = (Ct[RIP]) − (Ct[input] – log2 (input dilution factor)), ΔΔCt[RIP /Negative] = ΔCt [normalized RIP] - ΔCt [normalized Negative]. Fold Enrichment = 2^ (-ΔΔCt [RIP/Negative]).

### Polysome profiling analysis and RNA Sequencing

Isolation of the ribosome-protected fraction, library construction, high-throughput sequencing, and analysis were performed by Epibiotek Co.,ltd (Guangzhou, China). For Polysome profiling, U251 cells transduced with Lenti-NC and Lenti-Sh-NAT10 were treated with 50 µg/mL cycloheximide (CHX) for 15 min prior to harvest (*n* = 3 biological replicates per group). cells were treated with 100 μg/ml cycloheximide before lysis using the Epi™ Ribosome Profiling Kit (Epibiotek R1814). Ribosome-protected RNA fragments were isolated using RNA Clean&Concentrator-5 (ZYMO R1016), followed by rRNA depletion (Epibiotek R1805). Libraries were prepared and quality-checked using Qsep100 Analyzer (Bioptic). For RNA-seq, stranded mRNA libraries were prepared using VAHTS Kit (Vazyme NR612-02) and sequenced by Epibiotek Co., with subsequent bioinformatics analysis. The translation efficiency (TE) was calculated by TE =(FPKM in Ribo-seq) /(FPKM in RNA-seq). The results of polysome seq was shown in Supplementary Table 2.

### T7 biotin labeled BOC mRNA probe synthesis assay

The myc-T7-BOC plasmids were digested by Xbal and BstBI enzymes to obtain the linearization T7-BOC template. The digested product was subjected to 1% agarose DNA gel electrophoresis and the purified DNA template was obtained using the FastPure Gel DNA Extraction Mini Kit (Vazyme; Cat# DC301). DNA templates of the BOC mRNA probes were transcribed in vitro through the RiboTM RNAmax-T7 Biotin Labeled Transcription Kit (Ribobio; Cat# C1102) based on the manufacturer’s instructions.

### RNA pulldown assay

The BOC mRNA pulldown assays were performed using the highly efficient Magnetic RNA Pulldown Kit (Geneseed; Cat#P0201). To achieve a biotinylated mRNA probe, a biotin RNA Labeling Mix and T7 RNA polymerase were employed for transcription. The purified biotinylated mRNA underwent a heating process, followed by annealing to establish the appropriate secondary structure. Subsequently, the biotinylated mRNA probe was mixed with streptavidin-agarose beads for 1 hour at RT and allowed to interact. Afterward, the mRNA probes-beads complexes were combined with cell lysates for 2 hours at 4 °C. Finally, the enriched proteins were successfully separated via SDS-PAGE for subsequent analysis using WB.

### RNA denaturation gel electrophoresis assay

Briefly, 0.5 g agarose powder was placed in a conical flask filled with 36.5 mL DEPC water, followed by heating until the agarose was completely dissolved. After allowing the mixture to cool to approximately 50 °C, 5 mL of 10×MOPS (Solarbio; Cat#M1040), 8.5 mL of 37% formaldehyde (Macklin; Cat#F809702), and 5 μL RNA dye (Solarbio; Cat#SY1040) was added to denature the RNA. Finally, the 1% agarose denaturation gel was manufactured. Subsequently, 2 μg of isolated RNA was resuspended in a denaturation loading buffer (200 μL 10×MOPS, 270 μL 37% formaldehyde, 660 μL formamide (CSNpharm; Cat#CSN21440)). The mixture was maintained at a consistent temperature of 60°C for 10 minutes, followed by rapid cooling on ice. Subsequently, a 2× RNA denaturation loading buffer (Solarbio; Cat#R1055) was incorporated into the prepared mixture. For RNA labeling size determination, the ssRNA Ladder (BioLabs; Cat#N0362S) was used. The RNAs were separated on 1% agarose denaturing gels in 1×MOPS buffer. Electrophoresis was carried out for 45 minutes at 120 V. Lastly, the chemiluminescence image analysis system (Tanon; Shanghai) was employed to capture the image of the experimental results.

### ac4C dot blot assay

Total RNA was first extracted with TRIzol reagent, followed by poly(A)+ mRNA purification using oligo(dT)-conjugated magnetic beads (Beaver; Cat#70430-1). The mRNA quality was subsequently verified by dot blot hybridization. A mRNA sample (2–5 μg) was taken from the refrigerator and placed on an ice pack. Then 2 μL of RNA was dripped into the nylon membrane (Solarbio; Cat# YA1760). The plate with membrane was transferred to the chamber of SG Linker (with 254 nm bulb). The plate with the membrane was placed in the SG Linker chamber (which has a 254 nm bulb). Then, the cover was removed, and 125 mJ/cm^2^ of 254 nm UV radiation was used to penetrate the RNA into the membrane. This was followed by blocking in 10 mL of 3% BSA at RT for 1 hour. Afterward, the membrane was incubated overnight at +4 °C with the rabbit monoclonal ac4C antibody (Abcam; Cat# ab252215). Subsequently, the membrane was incubated for 1 hour at RT with anti-rabbit IgG-HRP (Proteintech; Cat#SA00001-2). Then, it was observed using the ECL substrate (UElandy; Cat#S6009) via an image analysis system (Tanon; Shanghai). The membrane was stained with 0.02% Methylene blue (Solarbio; Cat#G1301) for 30 minutes to detect internal controls before being rinsed with ddH2O. Finally, an image of the membrane was captured.

### mRNA stability assay

Target cells were grown in a complete DMEM medium supplemented with 5 μg/mL actinomycin D (MCE; Cat#HY-17559) for specific time intervals, including 0, 3, 6, and 9 hours. At each designated time point, after the cells had been harvested, total RNA was obtained to subsequently facilitate real-time PCR analysis. The half-live of mRNA was measured from the initial and final amounts of RNA.

$$k=\frac{\mathrm{ln}(C0)-\mathrm{ln}(C9)}{t(9)-t(0)};{t}_{(1/2)}=0.693/k$$

### Intracranial mouse model

Female BALB/c nude mice aged five weeks (GemPharmatech, China) were used to establish intracranial xenograft glioblastoma (GBM) models. The U251 cell line was selected for these experiments. Based on prior vitro validation in U251 cells (Fig. S2A), shRNA-1 was chosen for NAT10 knockdown due to its consistent and stable knockdown efficiency, while cells transduced with a non-targeting shRNA served as the negative control. both expressing stable luciferase. Mice were randomized into different groups using a random number table. Five mice were included in each group to meet the minimum sample size requirement to perform an independent sample t-test and one-way ANOVA analysis. U251 cells were resuspended in pre-cooled PBS and a total of 2 × 10^5^ U251 cells in 5 μL PBS were carefully injected into the right frontal node of anesthetized nude mice using isoflurane anesthesia. The injection was accurately performed 2 mm lateral to the midline and 1 mm posterior to the bregma. The detail of establishing intracranial xenograft GBM models as previously described [45]. The tumor size was assessed using luciferase intensity measurements acquired with the PerkinElmer IVIS Lumina Series III system (PerkinElmer, USA). They were humanely euthanized through the cervical dislocation method once the nude mice exhibited abnormal activities. Upon completing the experiments, the brains were extracted and preserved in 4% PFA for subsequent HE stains and immunohistochemical analysis. Both group allocation and outcome assessment were performed under blind conditions, where investigators and animal caretakers were unaware of treatment assignments throughout the experiment and data analysis.

### Statistical analysis

Statistical analysis was performed by a researcher who was blinded. All data are presented as mean ± standard error (SEM) of at least three independent experiments. GraphPad Prism 8 was applied to compare data between two groups employing the two-tailed unpaired Student’s t-test. To compare the means of three or more experimental subjects, the ANOVA analysis was performed. Log-rank tests were exploited to evaluate significant differences in Kaplan–Meier survival curves. The threshold of statistical significance was chosen at *p < 0.05, ** *p* < 0.01, *** *p* < 0.001, and ns *p* > 0.05.

## Supplementary information

Supplementary materials

WB original image

FigureS1

FigureS2

FigureS3

FigureS4

FigureS5

FigureS6

FigureS7

FigureS8

Supplementary Table 1

Supplementary Table 2

## Data Availability

All data generated or analysed during this study are available from the corresponding author on reasonable request.

## Code availability

Data processing and analysis were performed using R (version 4.2.1). The raw data were normalized using the limma package. Duplicate samples were removed based on unique patient identifiers. Sample names across different datasets were matched and harmonized using the dplyr and stringr packages. Differential expression analysis was conducted using the DESeq2 package. The custom R scripts used for data processing and analysis are available from the corresponding author upon reasonable request.
