# Supplementary material for: NAT10-mediated ac4C modifications regulate glioblastoma progression
Source: Cell Death Dis. 2026 Jan 8;17(1):181. doi: 10.1038/s41419-025-08315-3 (PMC12876961; doi:10.1038/s41419-025-08315-3)
Supplement: Supplementary file 1 — Supplementary materials [file 41419_2025_8315_MOESM1_ESM.docx]

**Figure S1.** (A) The expression of NAT10 in tumor and normal tissues in each malignancy was determined using data from the TCGA and GTEx datasets combined. (B) The expression level of NAT10 between HGG and LGG in the TCGA cohort, CGGA325 database and GSE108474 database. (C-G) Kaplan–Meier curves of glioma patients with two subgroups in the different tumor cohorts. (H) The expression of NAT10 was detected by WB in glioma and cancer adjacent tissues (CAT) (n = 30). (I) The levels of NAT10 were notably higher in patients with GBM cells than in normal human astrocyte cells (NHA). ns *p* > 0.05, * *p* < 0.05, * * *p* < 0.01, * * * *p* < 0.001.

**Figure S2.** (A-B) the protein level of NAT10 was significantly verified in U118 and U251 cells after NAT10 knockdown and overexpression. (C-H) NAT10 silencing led to an upregulation of the epithelial marker (E-cadherin) and downregulation of mesenchymal markers (N-cadherin and Vimentin) and the proliferation marker (PCNA). (I) Silencing NAT10 inhibits EMT, as evidenced by the altered expression of markers (E-cadherin, N-cadherin, vimentin) and the change in cell morphology from a mesenchymal to an epithelial phenotype. ns *p* > 0.05, * *p* < 0.05, * * *p* < 0.01, * * * *p* < 0.001.

**Figure S3.** (A) The abundance of BOC was considerably elevated in high-grade gliomas (HGG) than in lower-grade gliomas (LGG) in the TCGA database. (B) Kaplan‒Meier survival curve of patients with overall gliomas in the low and high BOC expression group. (C-H) The expression of BOC in NAT10-knockdown and overexpressing stably transfected U251 and U118 cells. (I) The representative images of IHC showed the expression of BOC and NAT10 in the mouse xenograft group. ns *p* > 0.05, * *p* < 0.05, * * *p* < 0.01, * * * *p* < 0.001.

**Figure S4.** (A-B) the protein level of BOC was significantly verified in U118 and U251 cells after BOC knockdown and overexpression. (C-D) The CCK8 assay to detect the proliferation ability of BOC-knockdown and BOC overexpress stably transfected in GBM cells. (E-F) The Transwell migration assay and Wound healing assay to detect the migration ability of BOC-knockdown and BOC overexpressing stably transfected in GBM cells. ns *p* > 0.05, * *p* < 0.05, * * *p* < 0.01, * * * *p* < 0.001.

**Figure S5.** (A-C) CCK-8 assays, Wound healing assay and Transwell migration assay demonstrated that upregulation of NAT10 can increase proliferation capacities in GBM cells, which can be inhibited by BOC knockdown. (D) The protein level of BOC and NAT10 was significantly verified in GBM cells, and the representative images of migration and transwell assays upon rescue experiments. (E-G) CCK-8 assays, Wound healing assay and Transwell migration assay indicated BOC reconstitution in NAT10-deficient cells significantly restored the impaired proliferative and migratory capacities. (H) The protein level of BOC and NAT10 was significantly verified in GBM cells, and the representative images of migration and transwell assays upon rescue experiments. ns *p* > 0.05, * *p* < 0.05, * * *p* < 0.01, * * * *p* < 0.001.

**Figure S6.** (A) KEGG analysis of signaling pathways involved in BOC. (B) Validation of four signaling pathways after knocking down BOC in glioma cells. (C) Analysis of PPI Network Proteins in BOC and Hedgehog signaling pathways. (D) Validation of Hedgehog signaling pathways detected by WB.

**Figure S7.** (A) The protein expression of HIF-1α, NAT10, and BOC were detected with treating with CoCl_2_ (24h) for 0, 0.1, 0.2, 0.3, and 0.4 mM in U251 cells. (B) The protein expression of HIF-1α, NAT10, and BOC were detected with treating with CoCl_2_ (0.3 mM) for 0, 6, 12, 18, and 24h in U251 cells. (C) The mRNA level of HIF-1α, NAT10, and BOC were detected by RT-PCR treated by 0.3 mM CoCl_2_ for 24h in U251 cells. (D) The mRNA level of HIF-1α, NAT10, and BOC were detected by RT-PCR treated with normoxia and hypoxia condition in U251 cells. (E) BOC expression in HIF-1α-knockdown and NAT10-knockdown stably transfected U251 cells under the normoxia and hypoxia condition. (F) Immunofluorescent staining showing NAT10 was regulated by HIF1α under the hypoxia conditions. ns *p* > 0.05, * *p* < 0.05, * * *p* < 0.01, * * * *p* < 0.001.

**Figure S8.** (A) Dose-response curve of Remodelin in U251 cells, showing IC50 determination (24 h treatment). (B) Western blot analysis of NAT10 protein levels after Remodelin treatment (37μM, 24 h). (C) Dot blot assay demonstrating reduced ac4C modification of mRNA following Remodelin treatment (37μM, 24 h). (D) CCK-8 proliferation assay under hypoxia (1% O₂), revealing Remodelin-mediated suppression of cell growth. (E) The mRNA acetylation levels (ac4C), NAT10 and BOC protein expression after Remodelin treatment under hypoxia (1% O₂, 37 μM, 24 h). ns *p* > 0.05, * *p* < 0.05, * * *p* < 0.01, * * * *p* < 0.001.
