# Supplementary material for: NAT10-mediated ac4C modifications regulate glioblastoma progression
Source: Cell Death Dis. 2026 Jan 8;17(1):181. doi: 10.1038/s41419-025-08315-3 (PMC12876961; doi:10.1038/s41419-025-08315-3)

Figure 1

NAT10

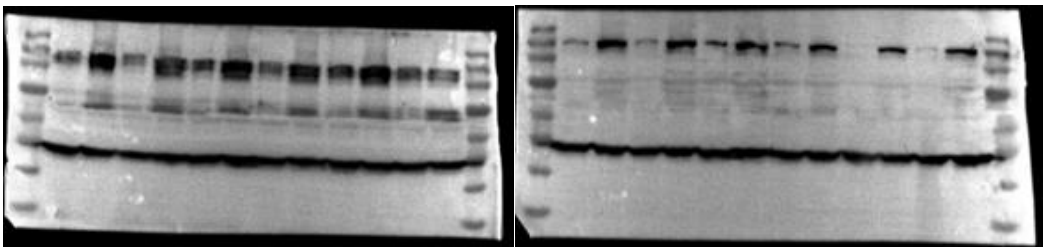

Figure 4F

118 NAT10

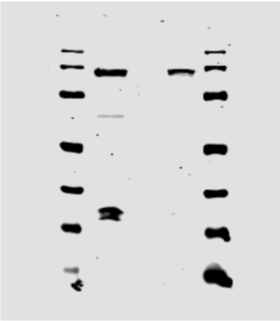

U251 NAT10

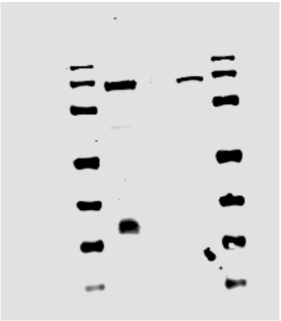

Figure 4H

U118 NAT10

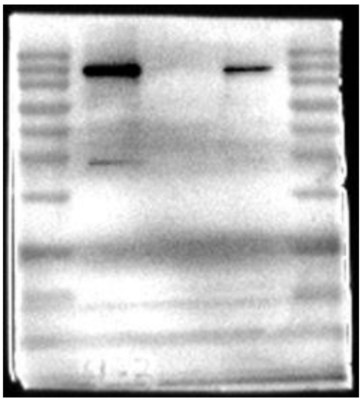

U251 NAT10

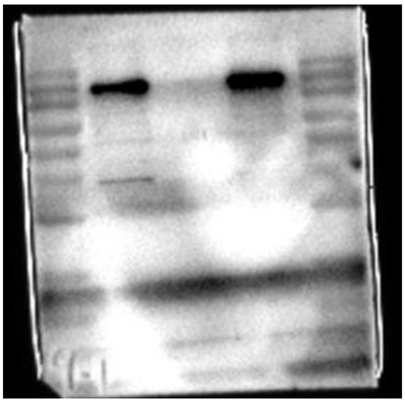

Figure 5F

U118 **U251**

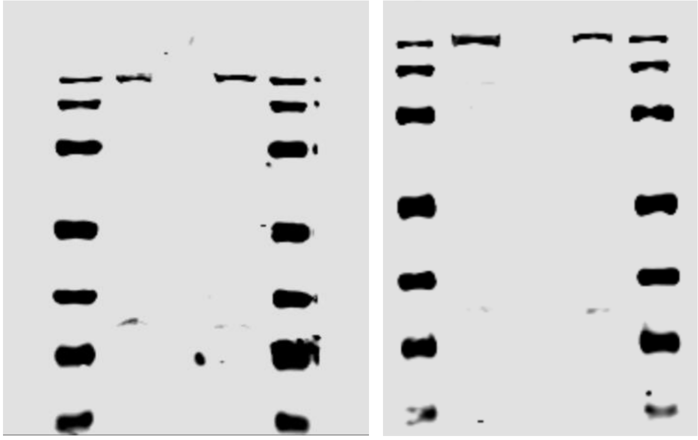

Figure 6G

118 BOC

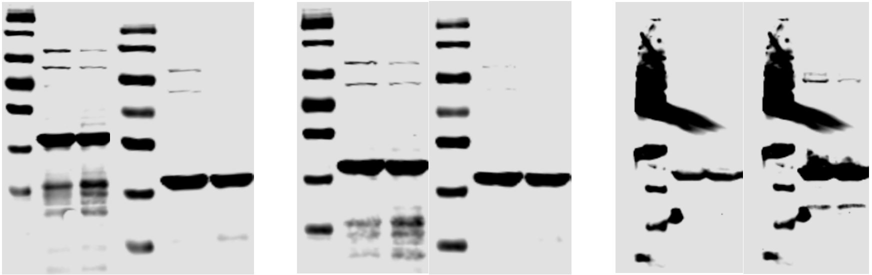

251 BOC

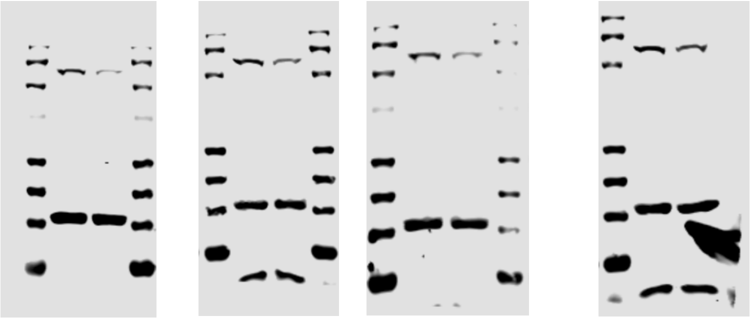

Figure 7D

HIF1A

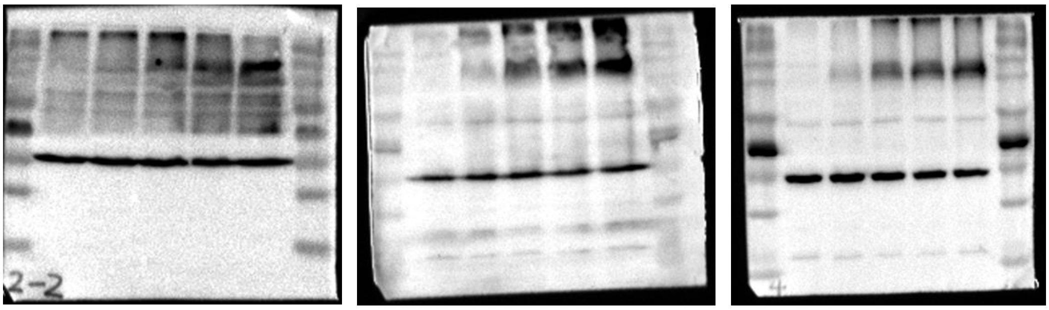

NAT10

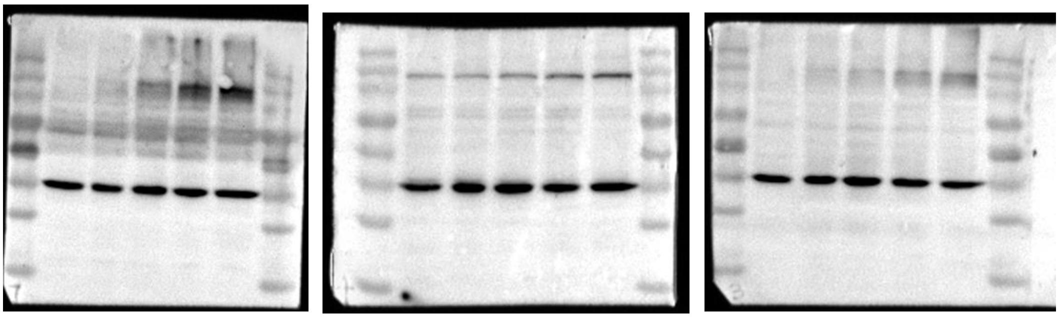

BOC

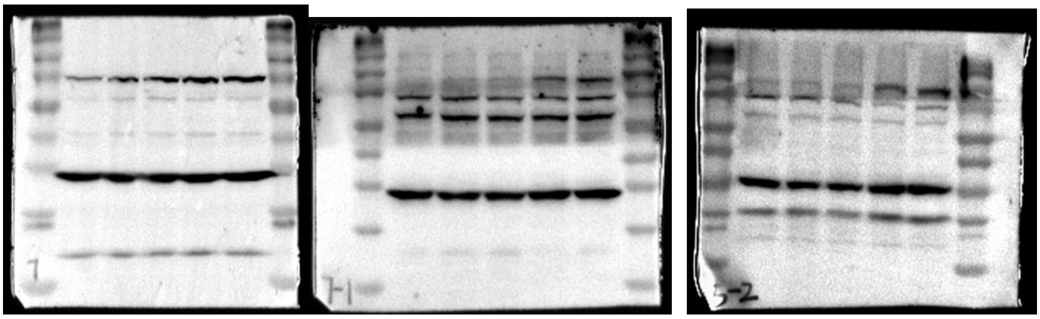

Figure 7E

HIF1A

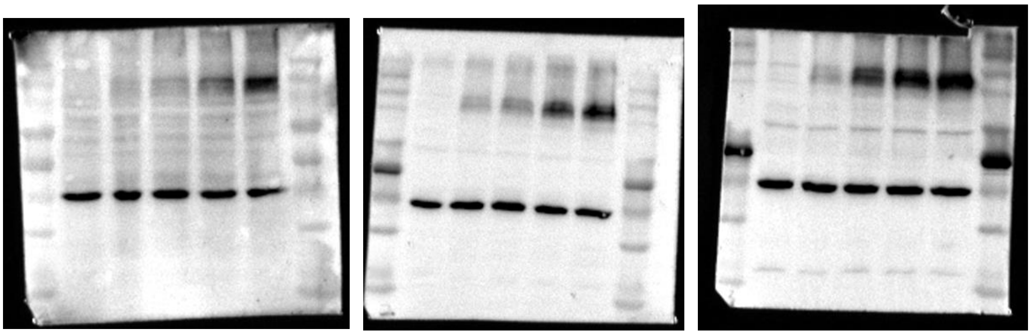

NAT10

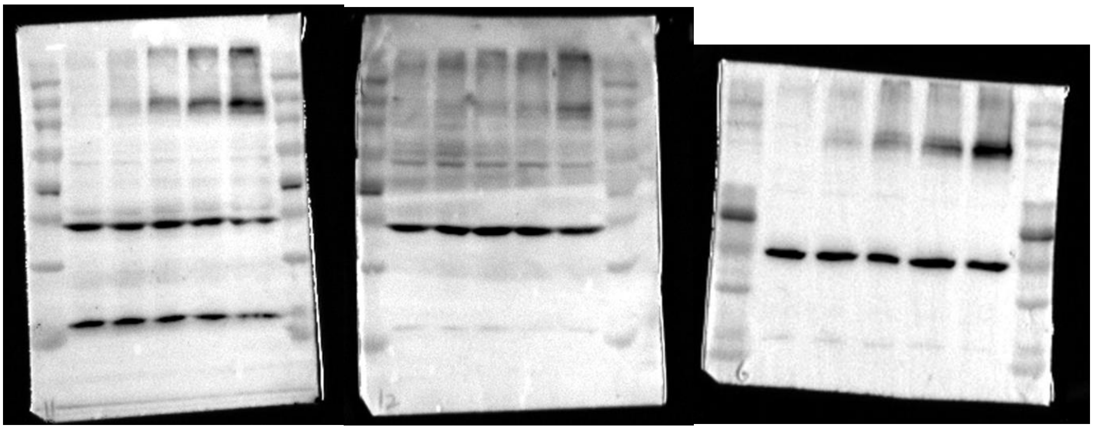

BOC

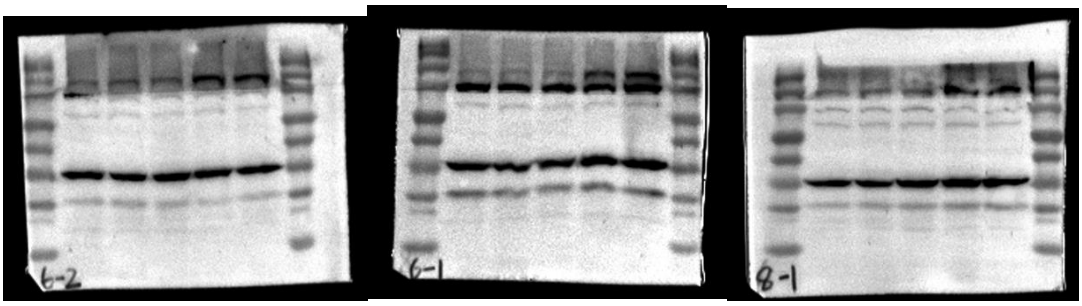

Figure 7H

HIF1A

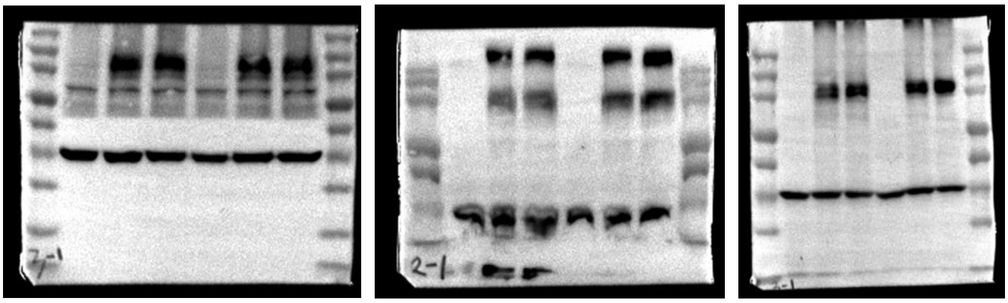

NAT10

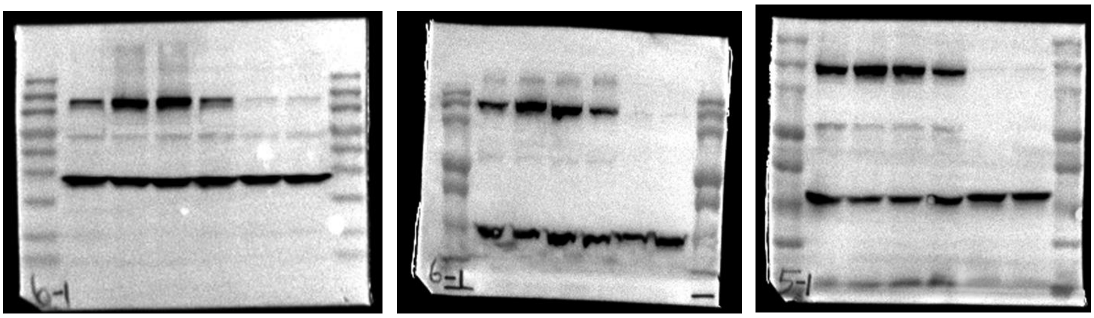

BOC

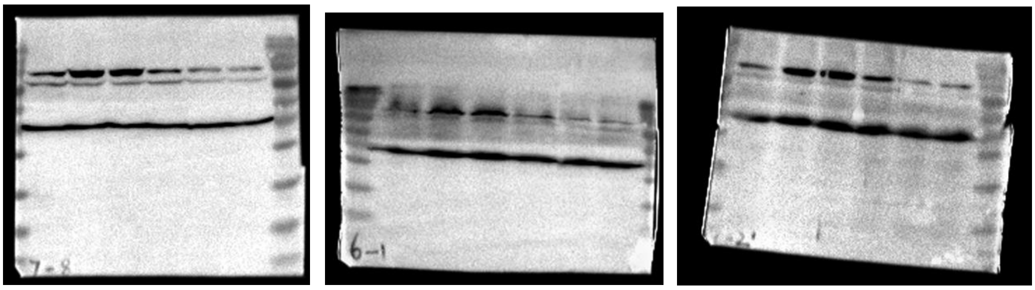

Figure 8

U118

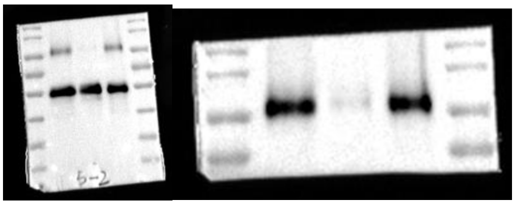

U251

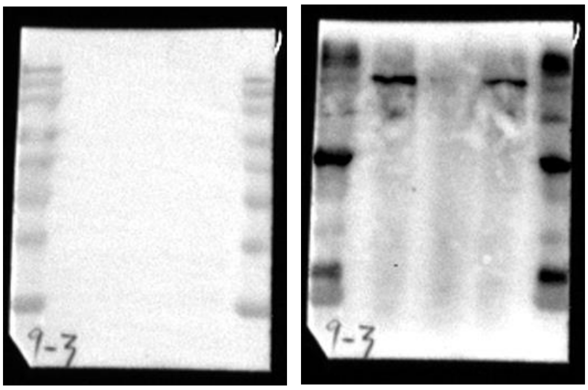

Figure S1H

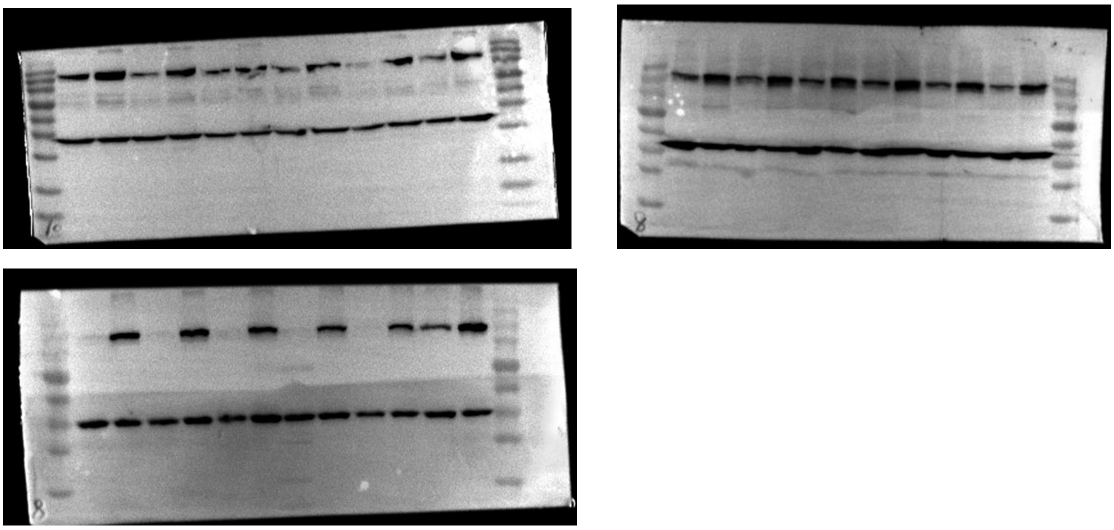

Figure S1I

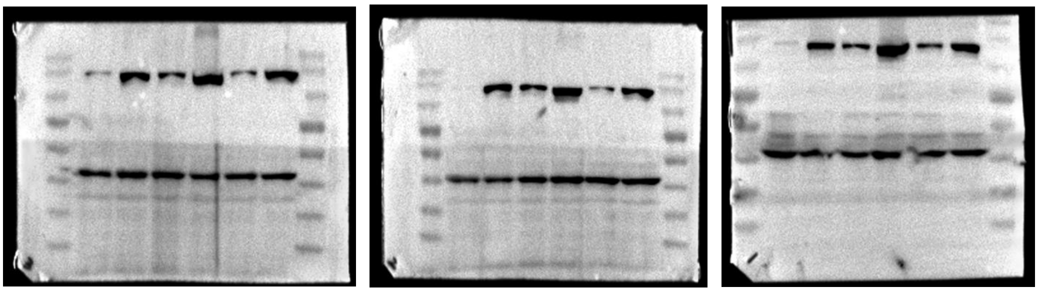

Figure S2A

U118 NAT10

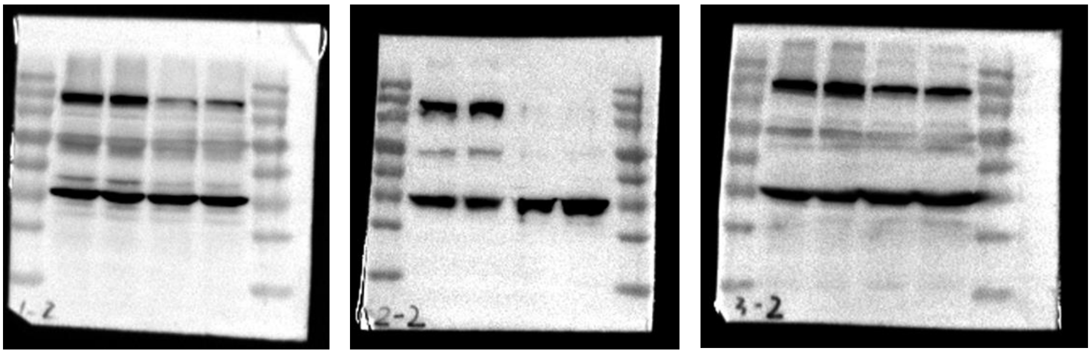

U251 NAT10

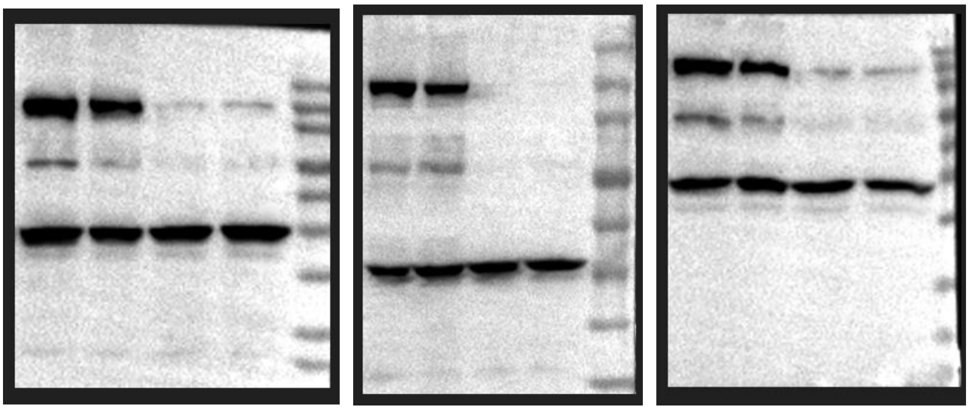

Figure S2B

U118 NAT10

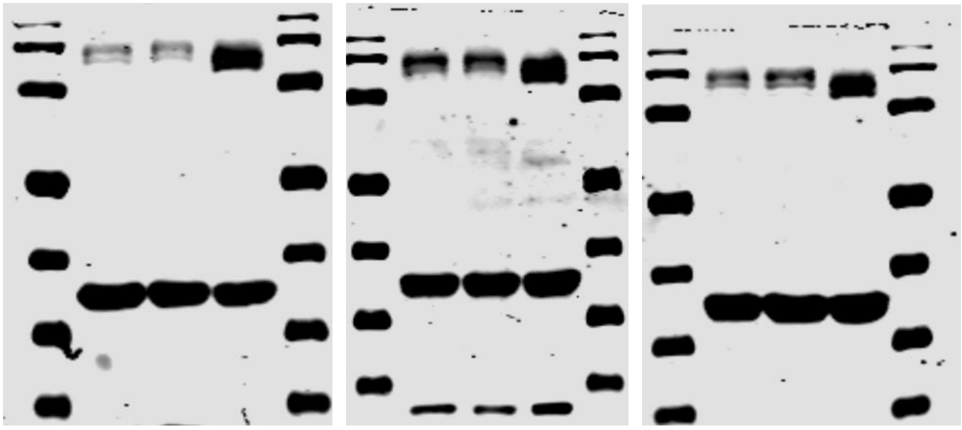

U251 NAT10

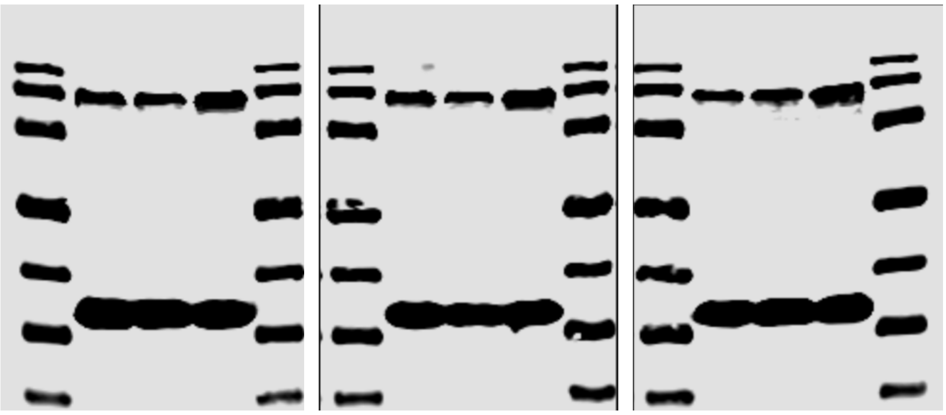

Figure S2C

Ecad (120 kda) /PCNA (36kda)/ACTIN(42kda)

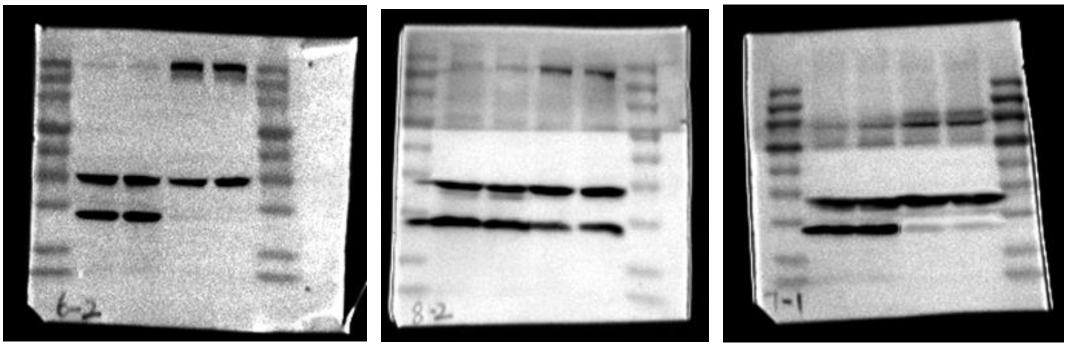

Ncad (120 kda) /VIM (54kda)/ ACTIN(42kda)

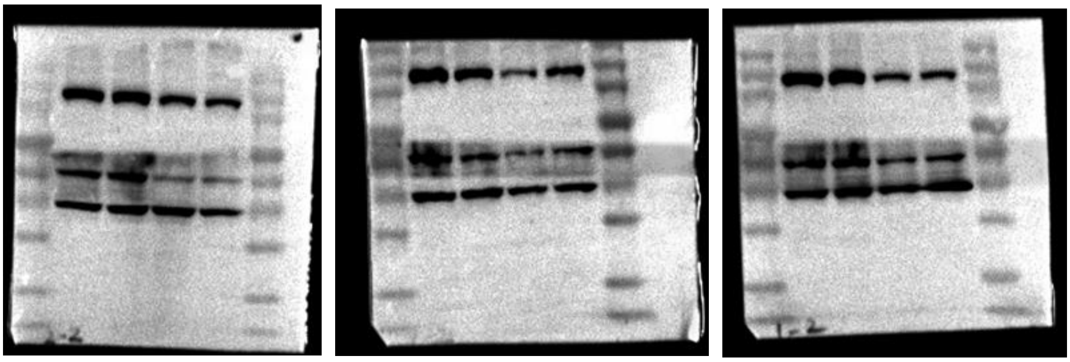

Figure S2F

Ecad (120 kda) /PCNA (36kda)/ACTIN(42kda)

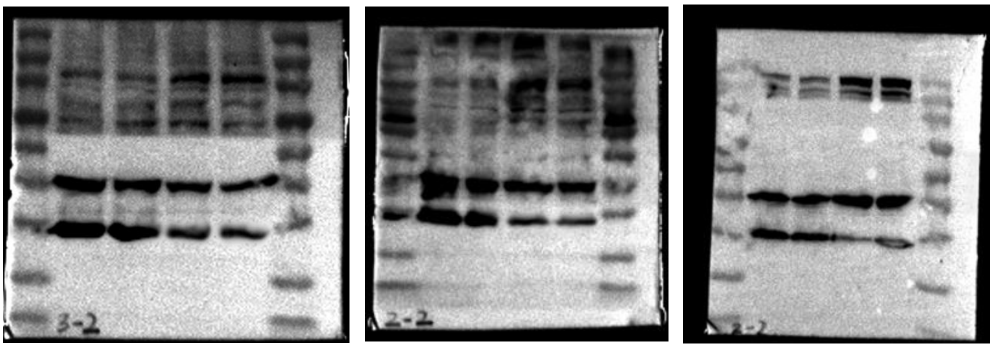

Ncad (120 kda) /VIM (54kda)/ ACTIN(42kda)

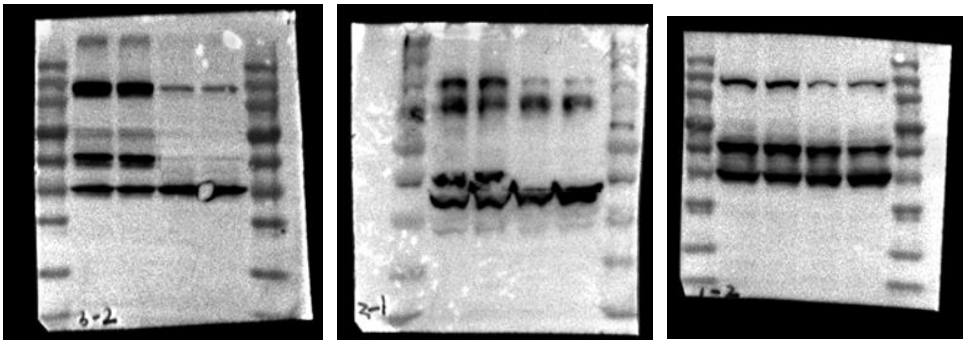

Figure S3D

U118 NAT10

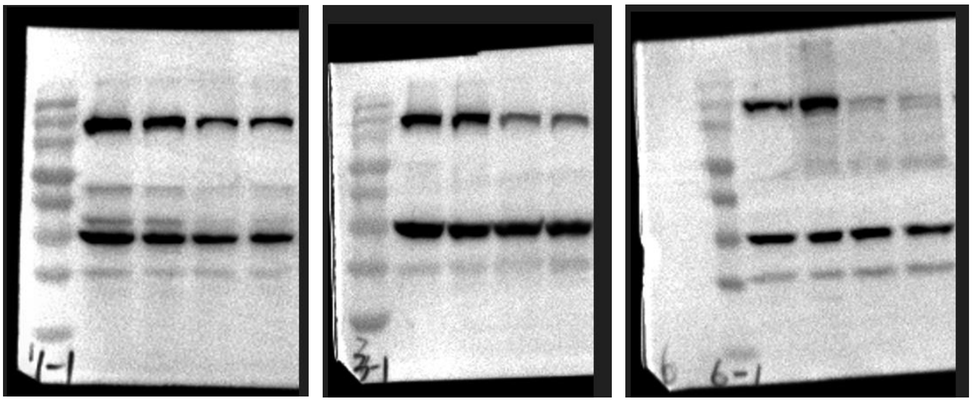

U118 BOC

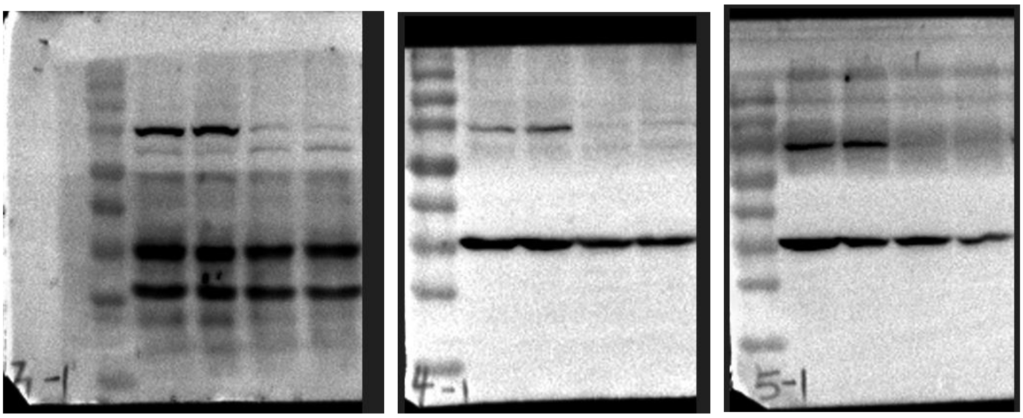

U251 NAT10

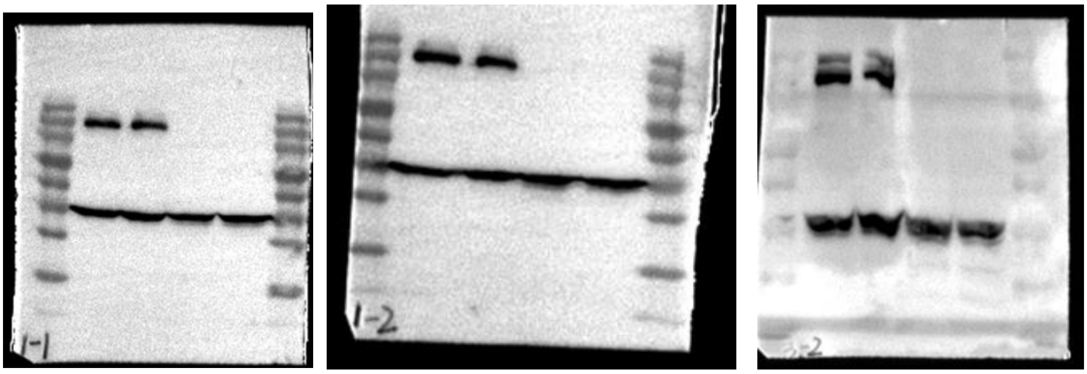

U251 BOC

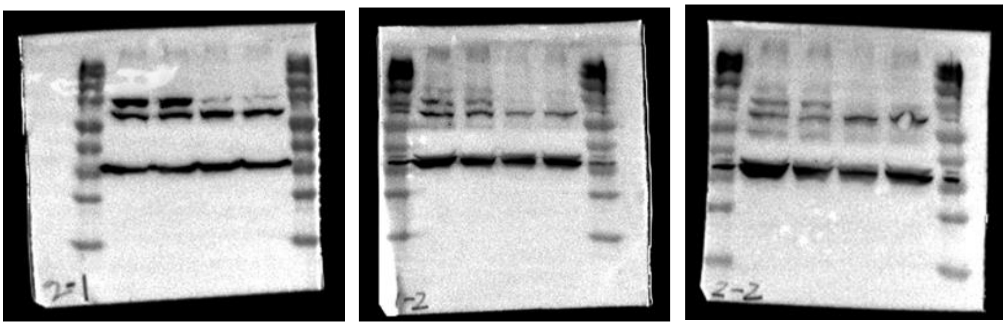

Figure S3G

U118 NAT10

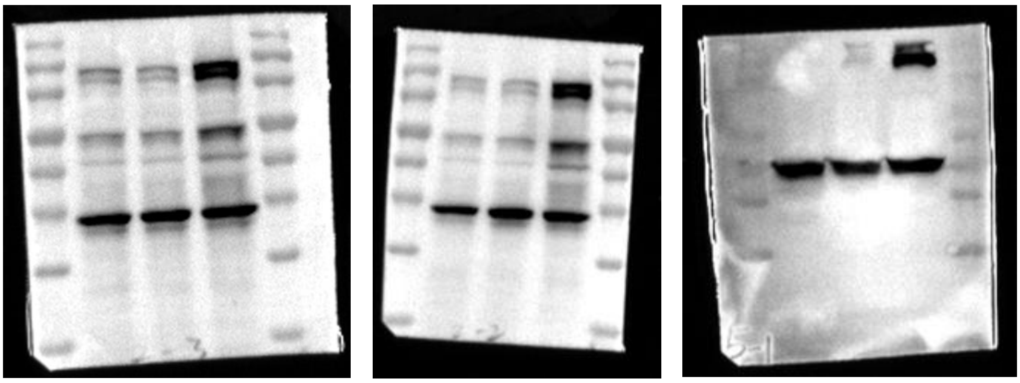

U118 BOC

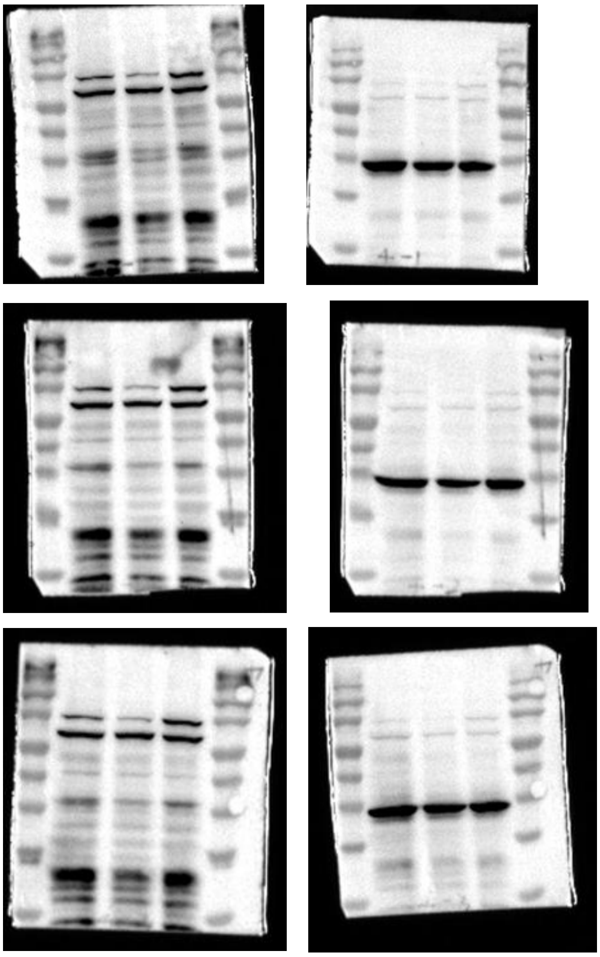

U251 NAT10

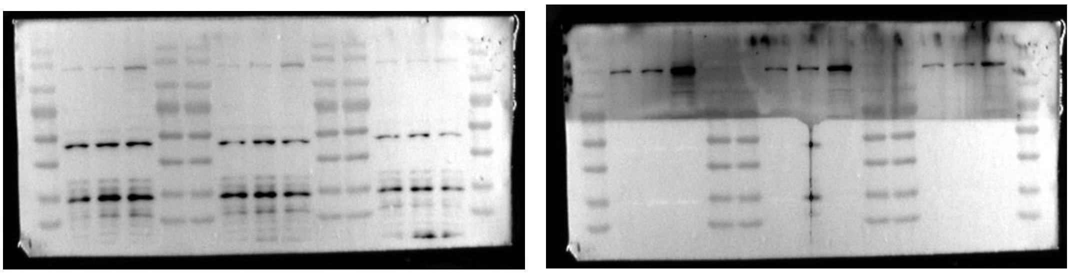

U251 BOC

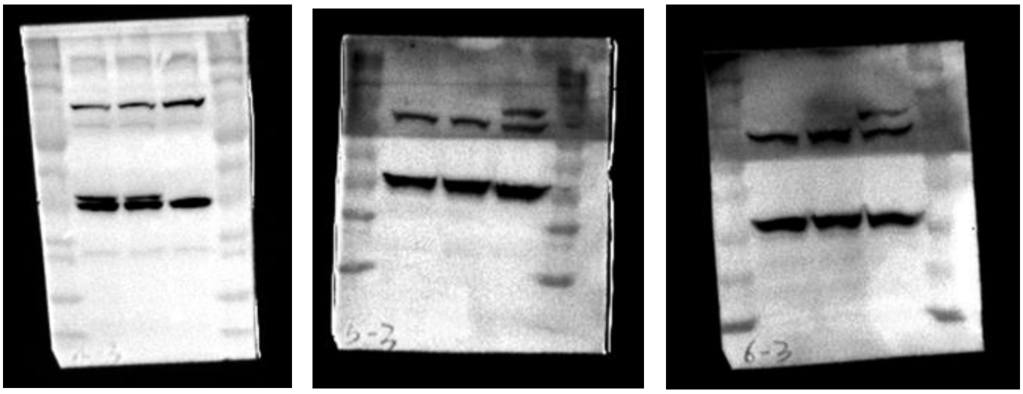

Figure S4A

U118 BOC

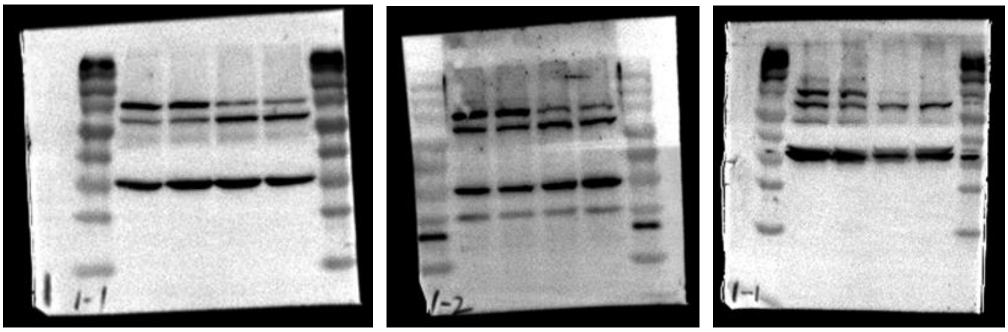

U251 BOC

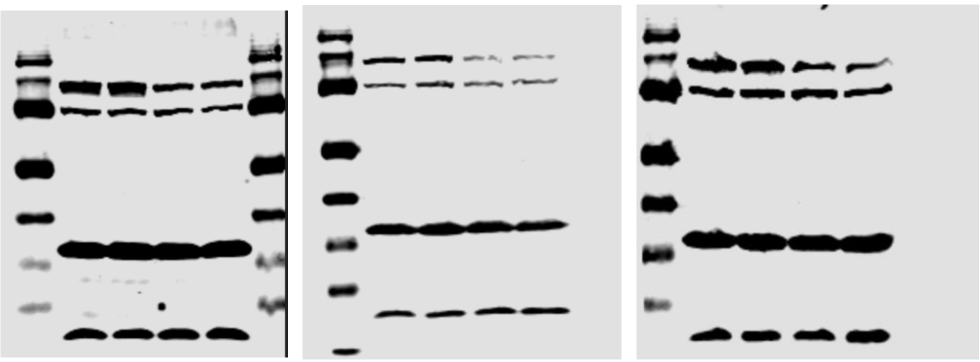

Figure S4B

U118 BOC

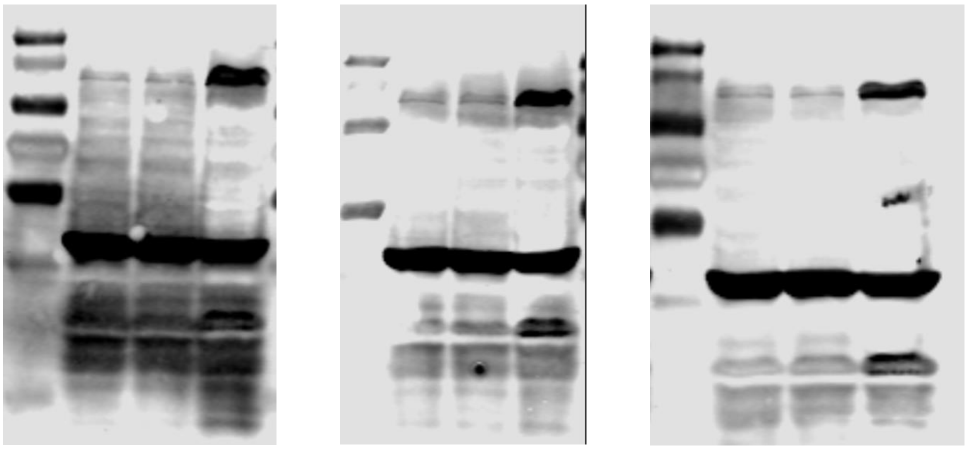

U251 BOC

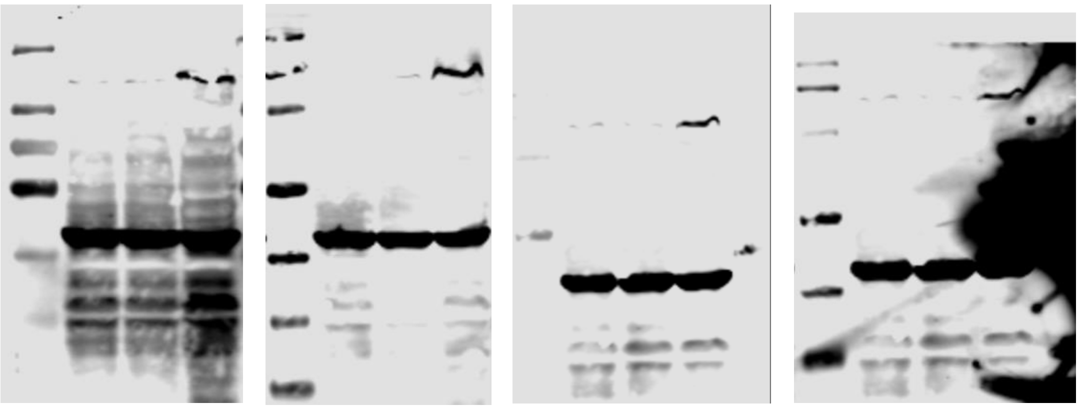

Figure S4G

U118 NAT10

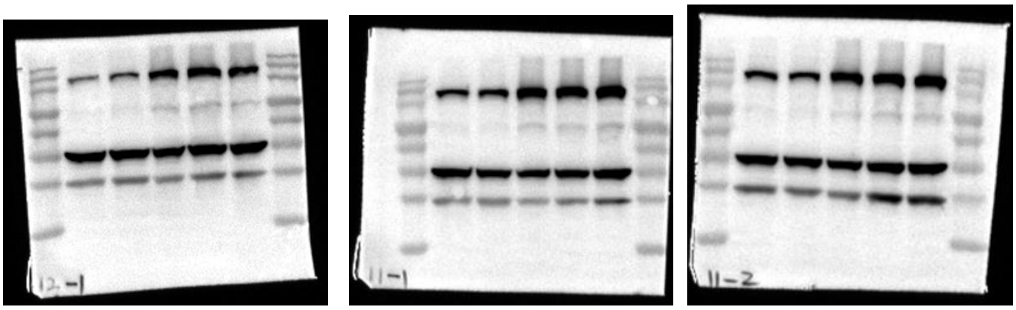

U118 BOC

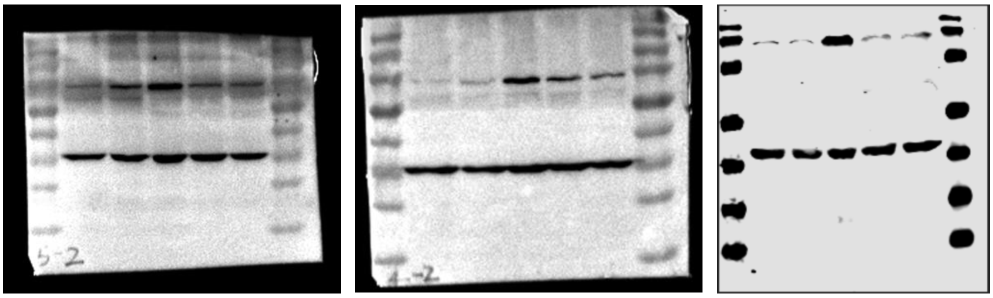

U251NAT10

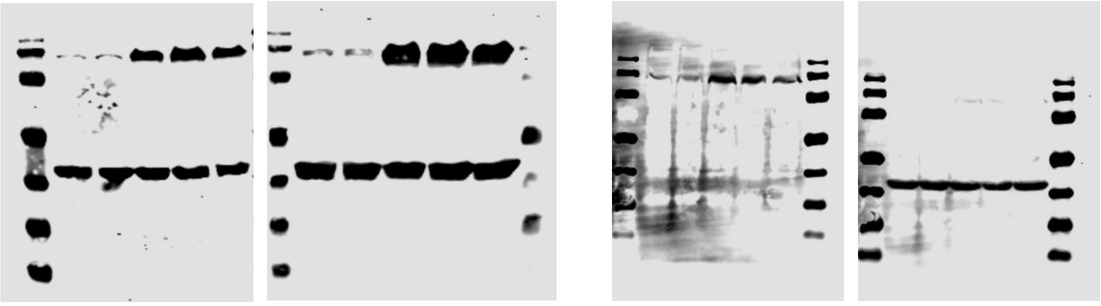

U251 BOC

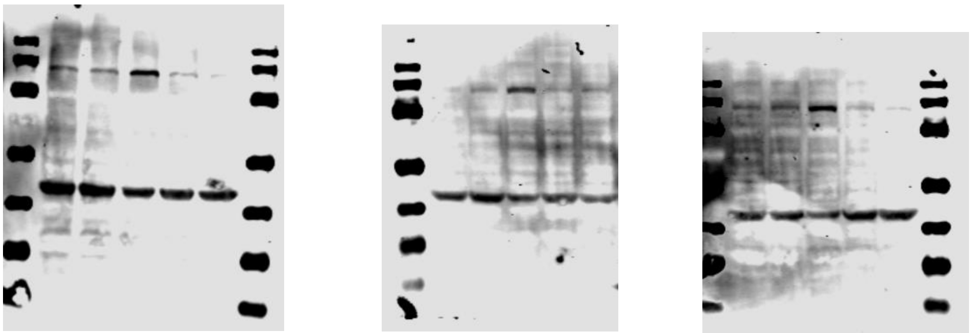

Figure S4H

U118 NAT10

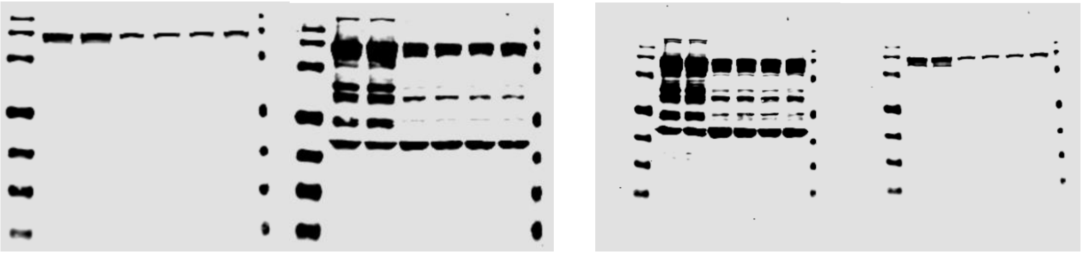

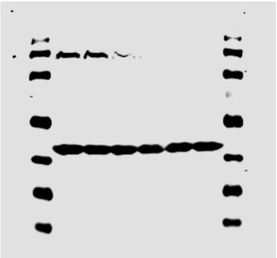

U118 BOC

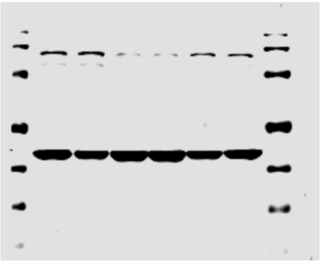

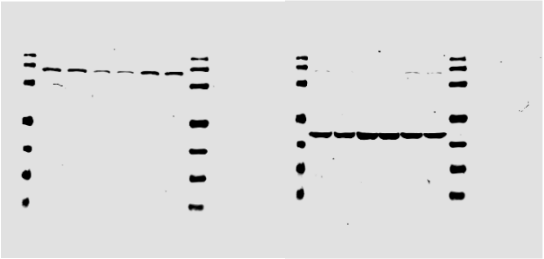

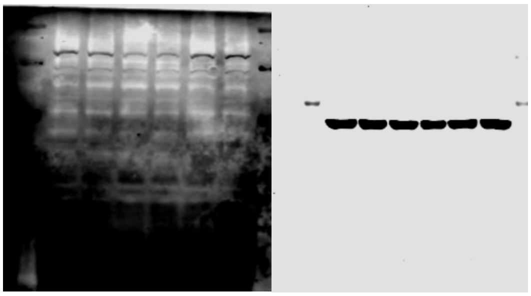

U251NAT10

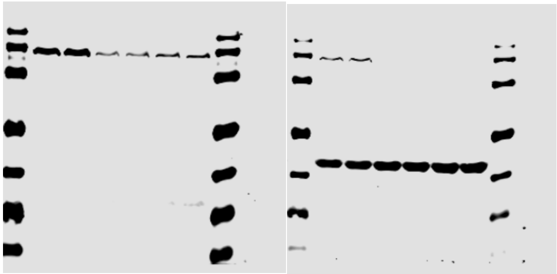

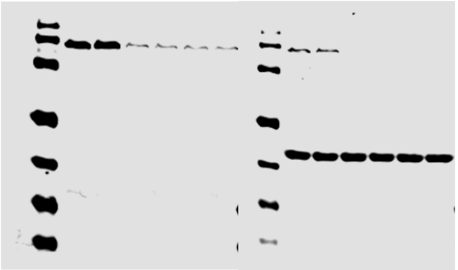

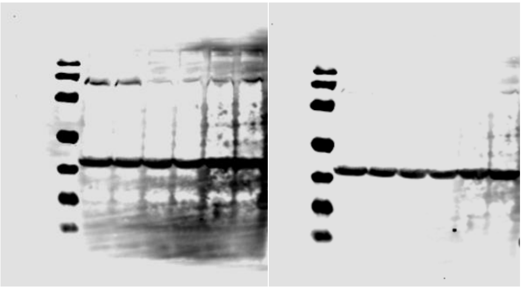

U251 BOC

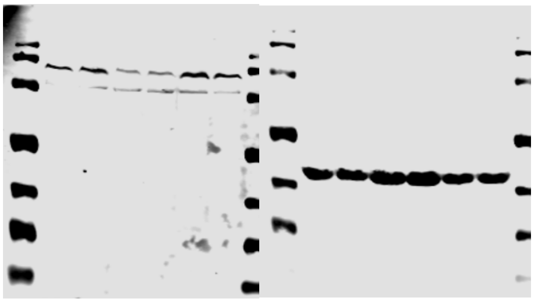

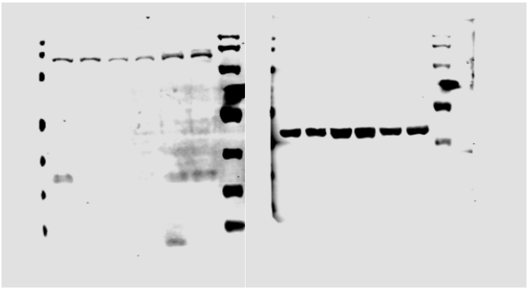

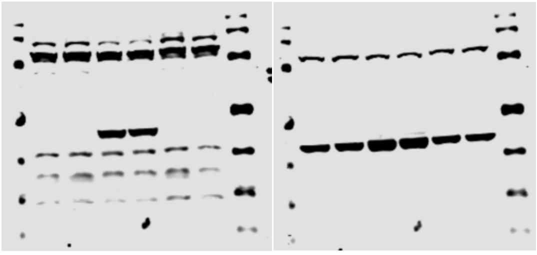

Figure S6D

GLI1

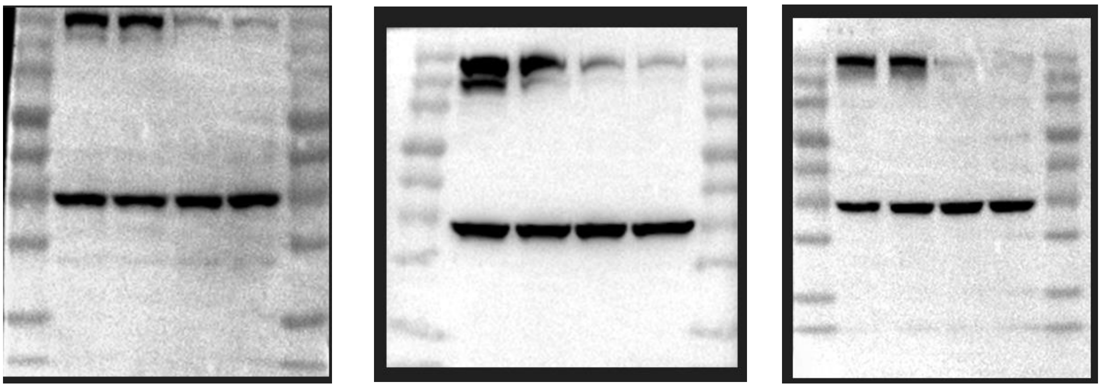

Figure S7A

HIF1A

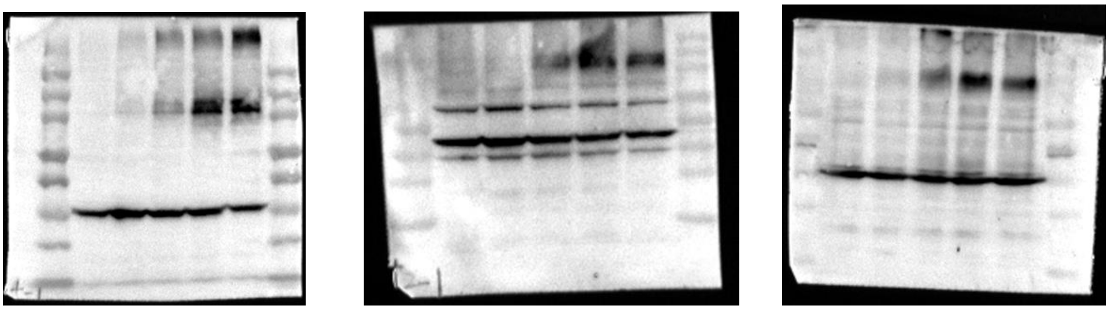

NAT10

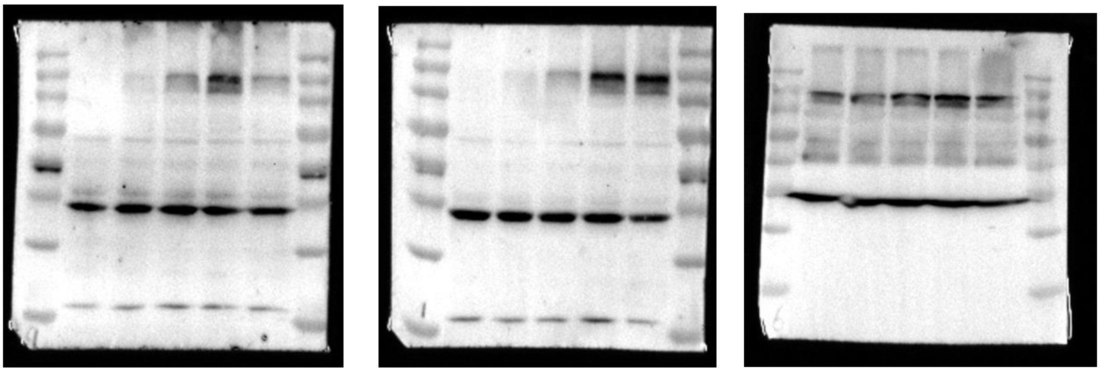

BOC

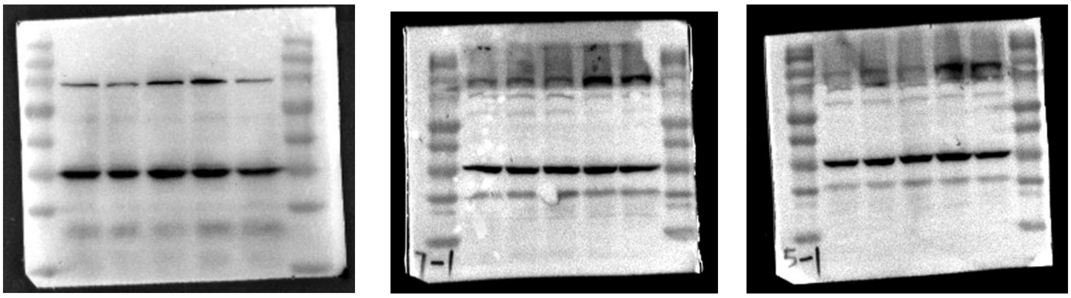

Figure S7B

HIF1A

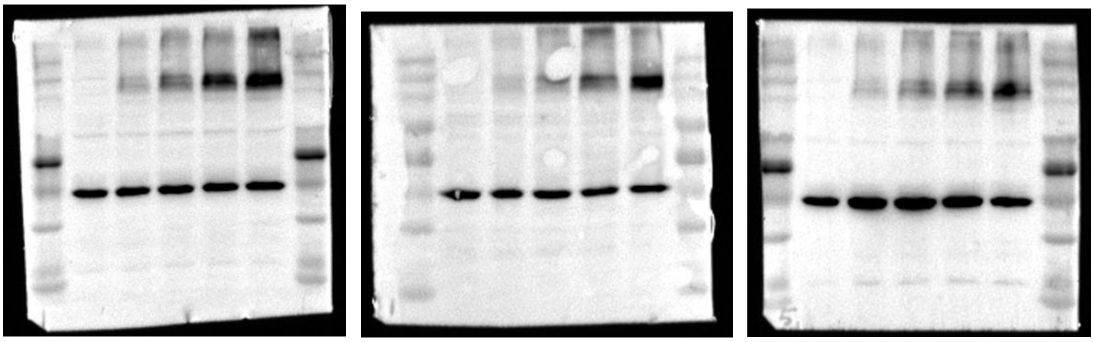

NAT10

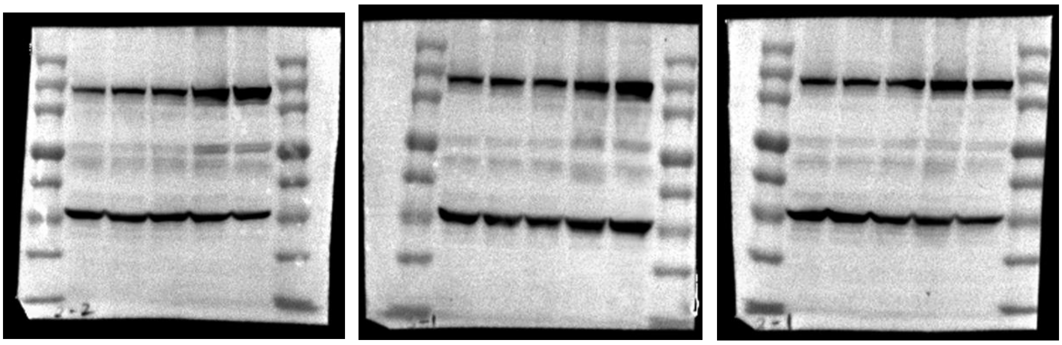

BOC

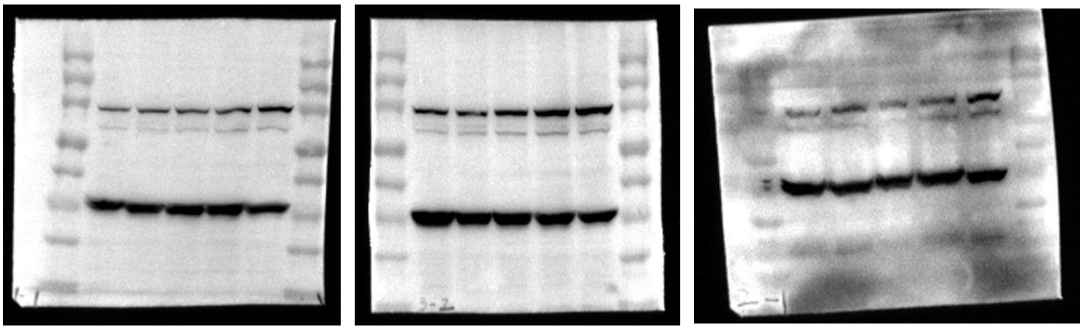

Figure S7E

HIF1A

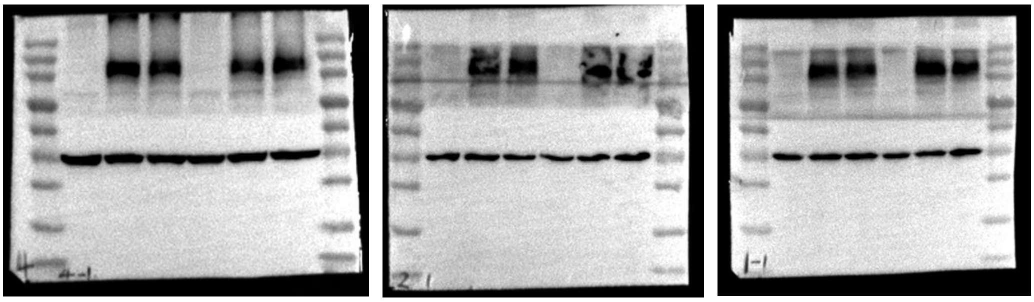

NAT10

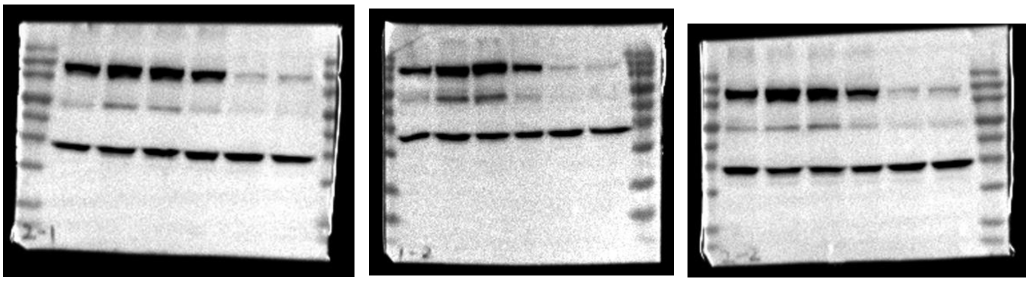

BOC

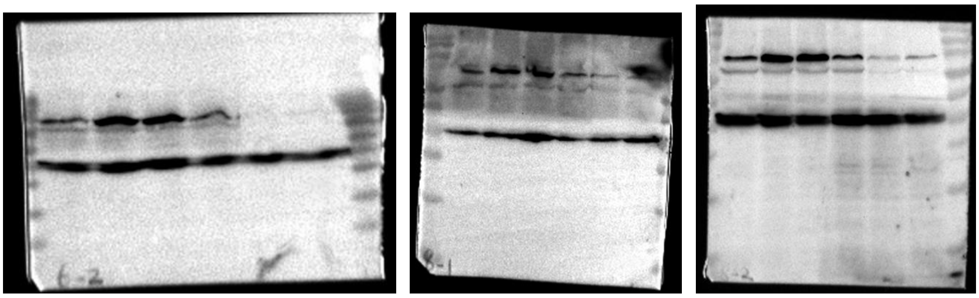

Figure S8B

NAT10

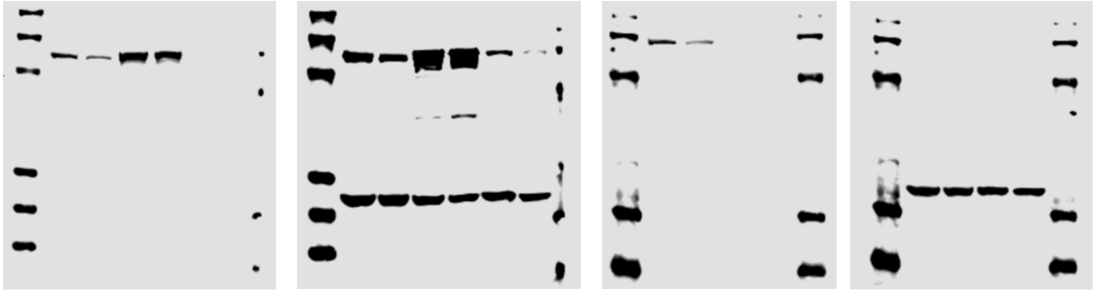

Figure S8E

NAT10

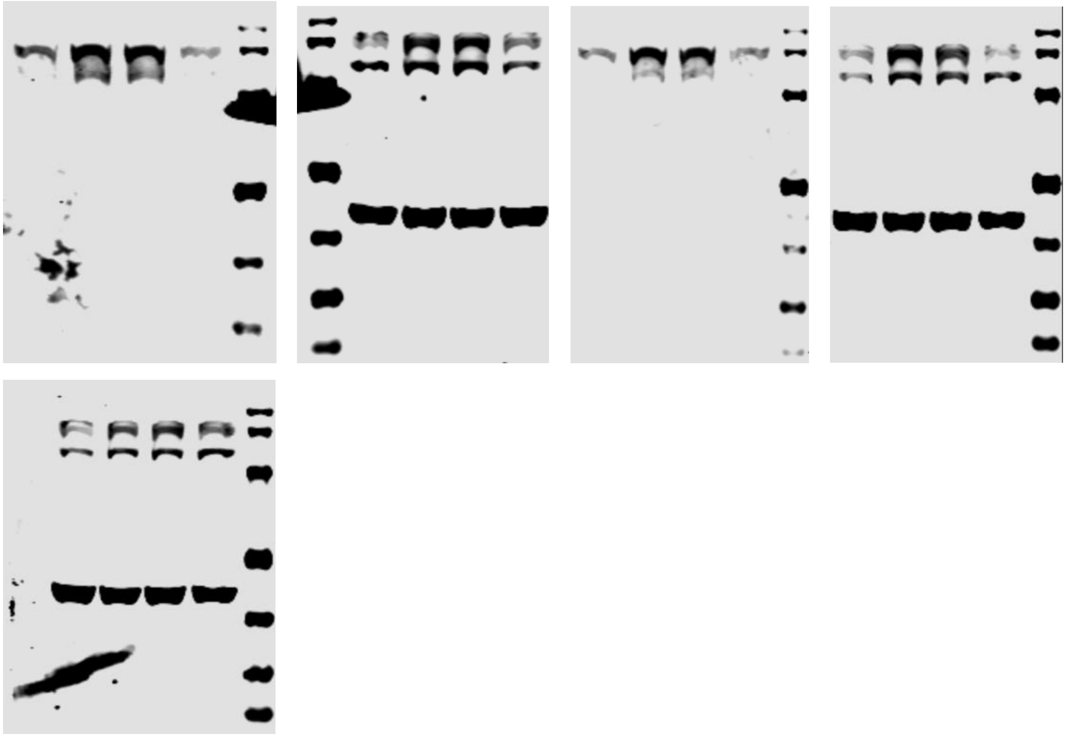

BOC

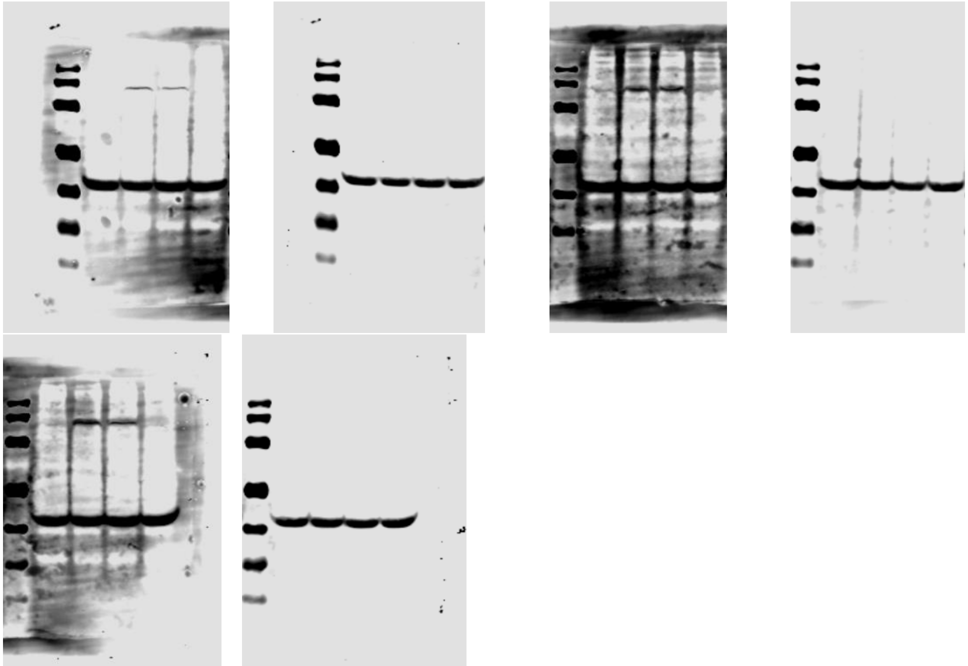

Supplement: Supplementary file 2 — WB original image [file 41419_2025_8315_MOESM2_ESM.pdf]
