# Supplementary figures and images for: NAT10-mediated ac4C modifications regulate glioblastoma progression
Source: Cell Death Dis. 2026 Jan 8;17(1):181. doi: 10.1038/s41419-025-08315-3 (PMC12876961; doi:10.1038/s41419-025-08315-3)

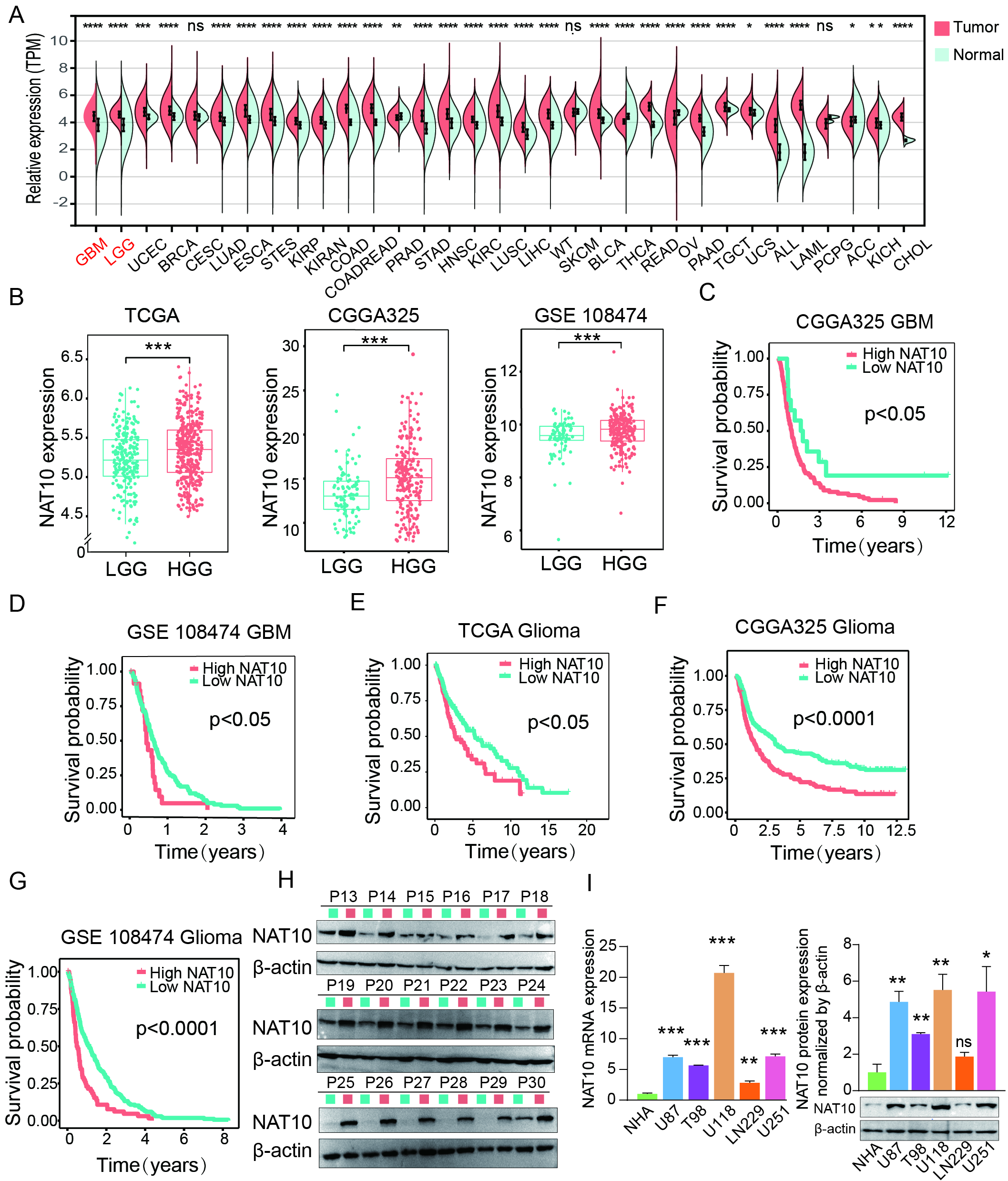

Supplement: Supplementary file 3 — FigureS1 [file 41419_2025_8315_MOESM3_ESM.tif]

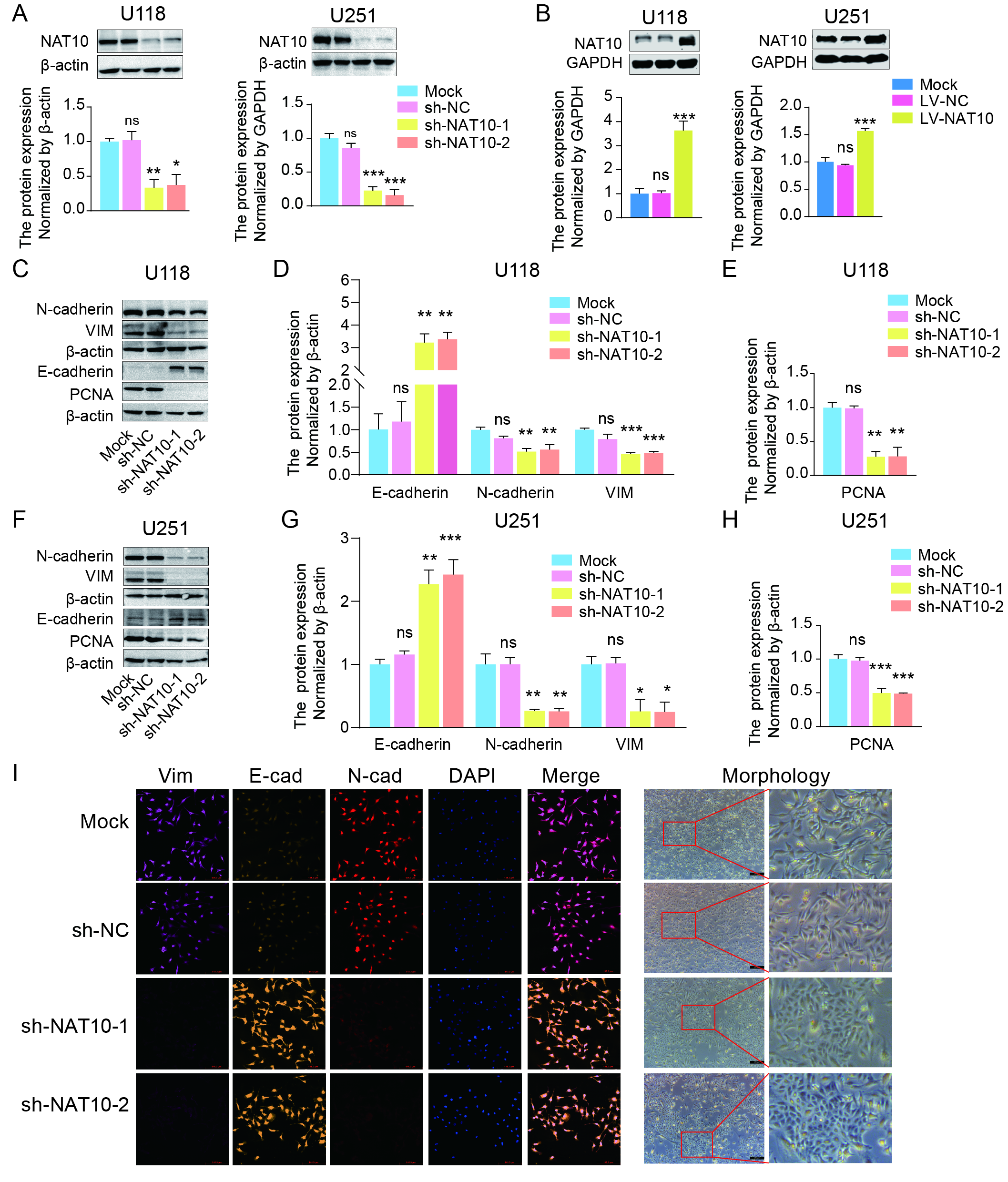

Supplement: Supplementary file 4 — FigureS2 [file 41419_2025_8315_MOESM4_ESM.tif]

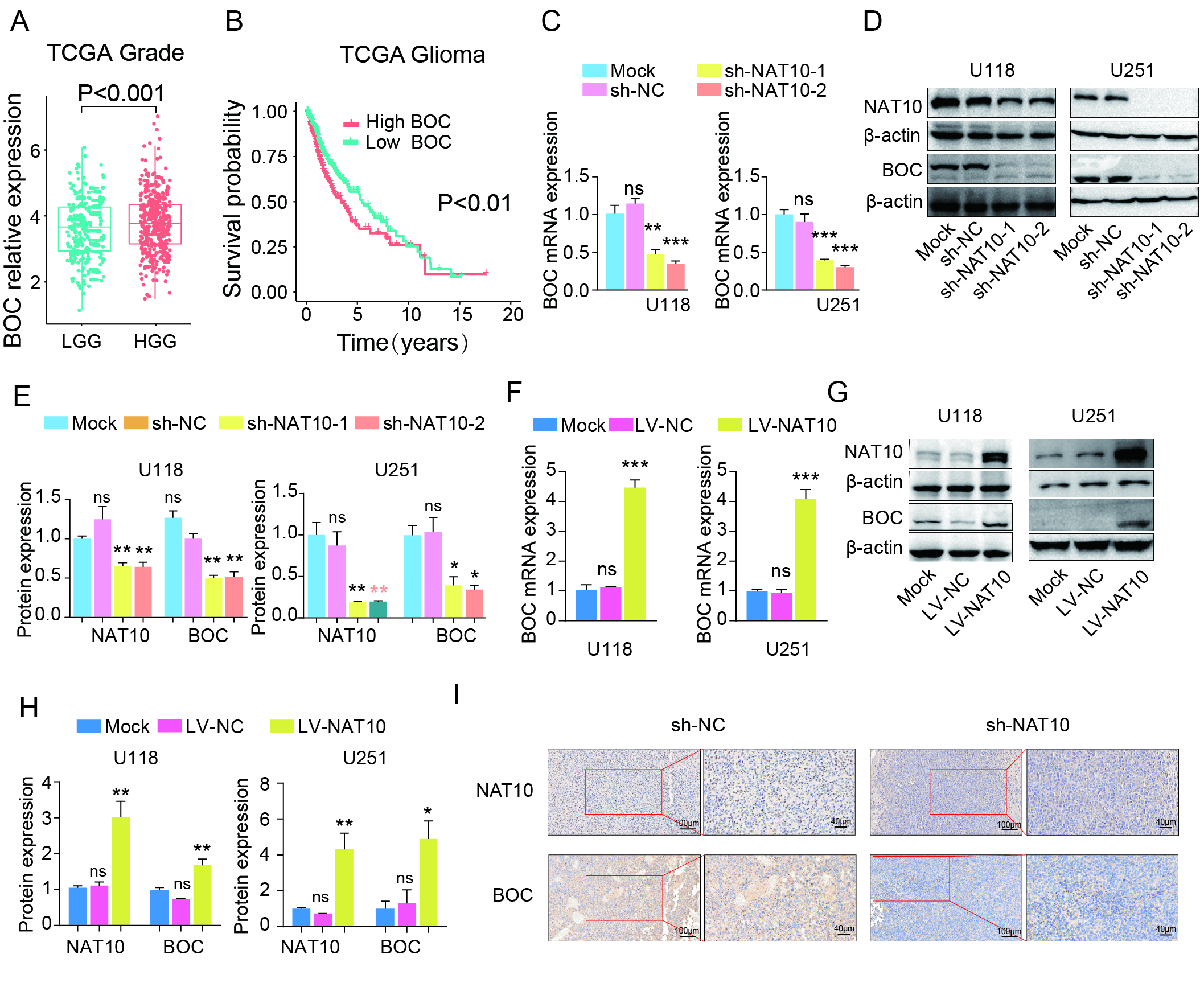

Supplement: Supplementary file 5 — FigureS3 [file 41419_2025_8315_MOESM5_ESM.tif]

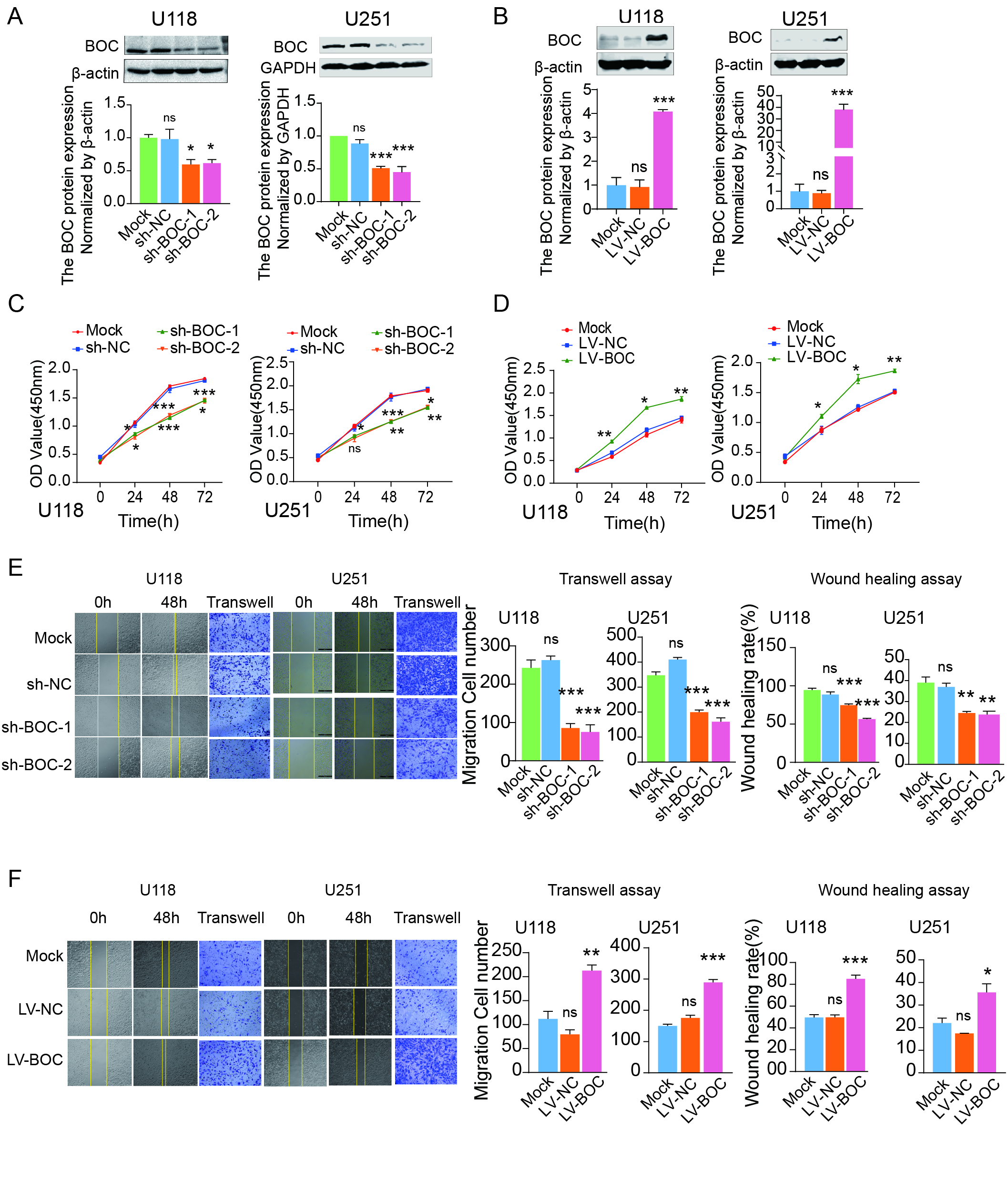

Supplement: Supplementary file 6 — FigureS4 [file 41419_2025_8315_MOESM6_ESM.tif]

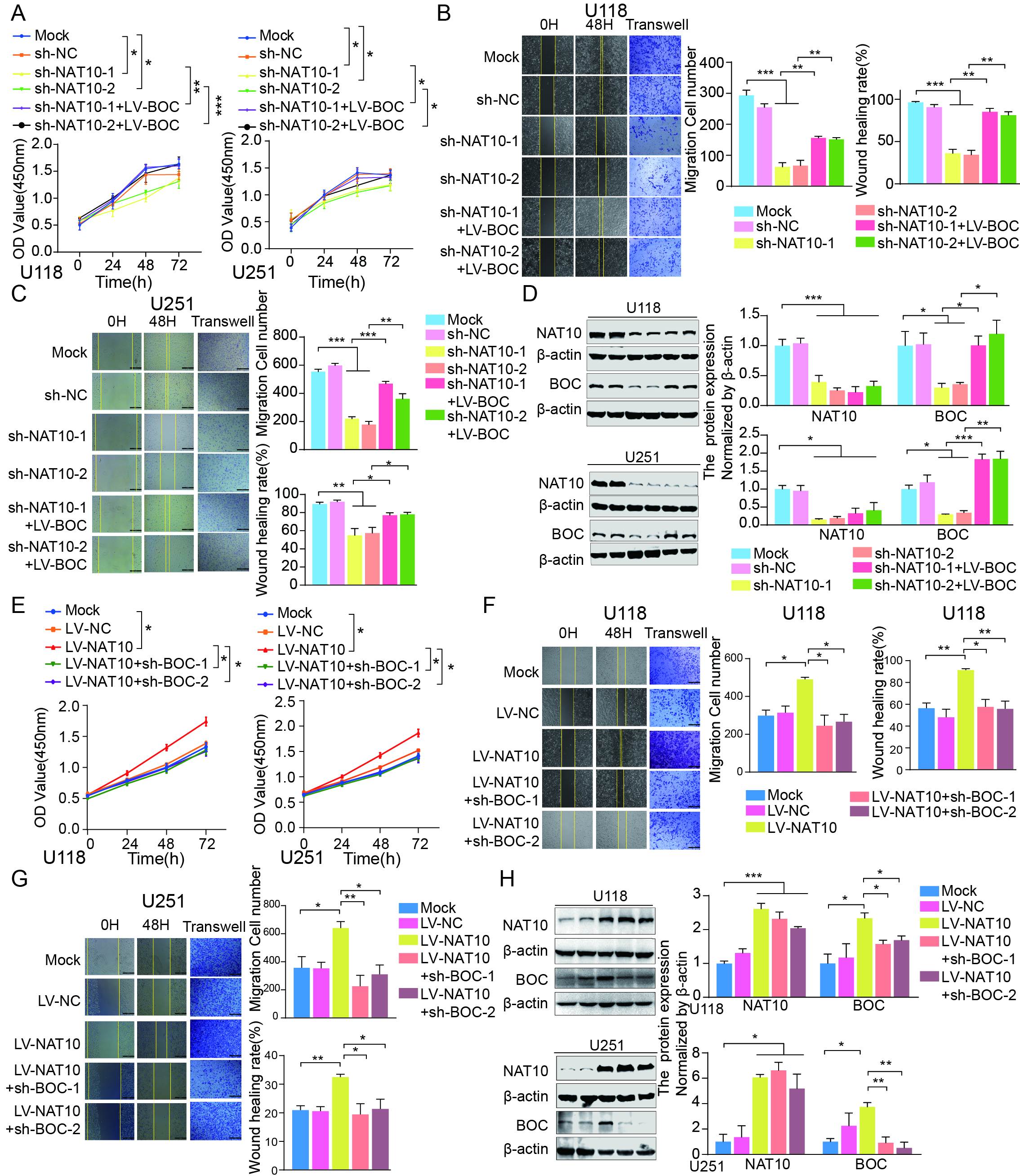

Supplement: Supplementary file 7 — FigureS5 [file 41419_2025_8315_MOESM7_ESM.tif]

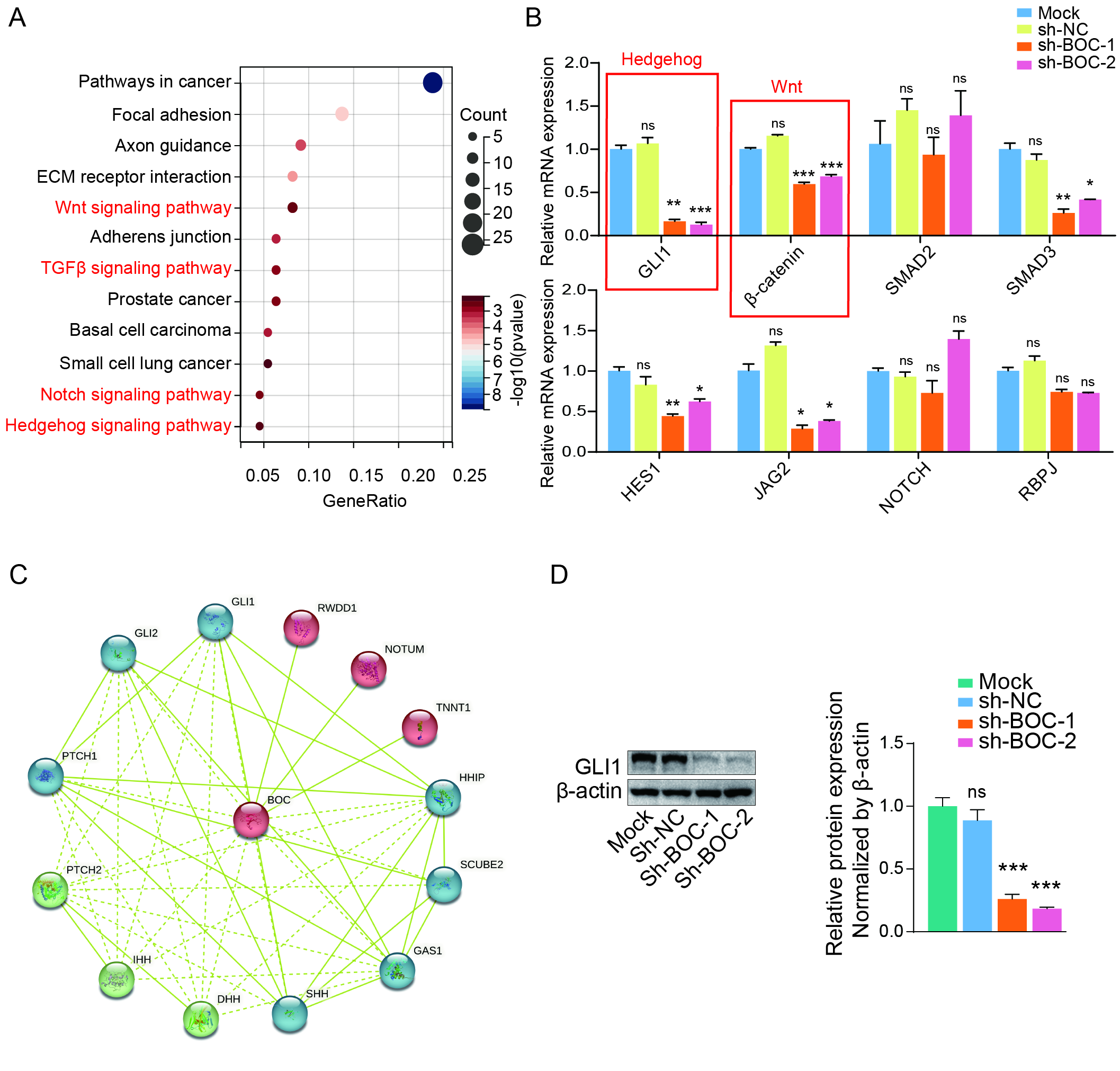

Supplement: Supplementary file 8 — FigureS6 [file 41419_2025_8315_MOESM8_ESM.tif]

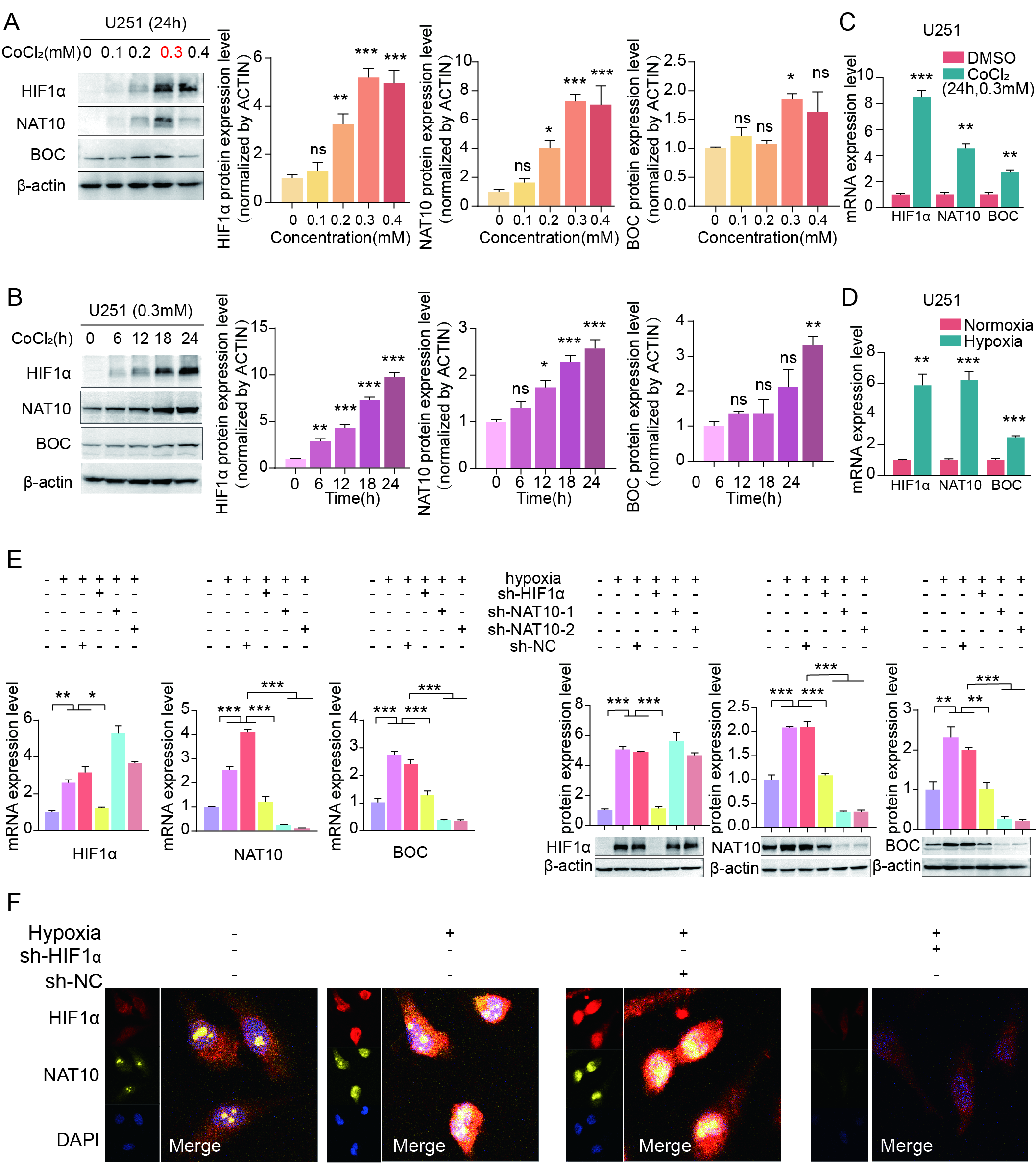

Supplement: Supplementary file 9 — FigureS7 [file 41419_2025_8315_MOESM9_ESM.tif]

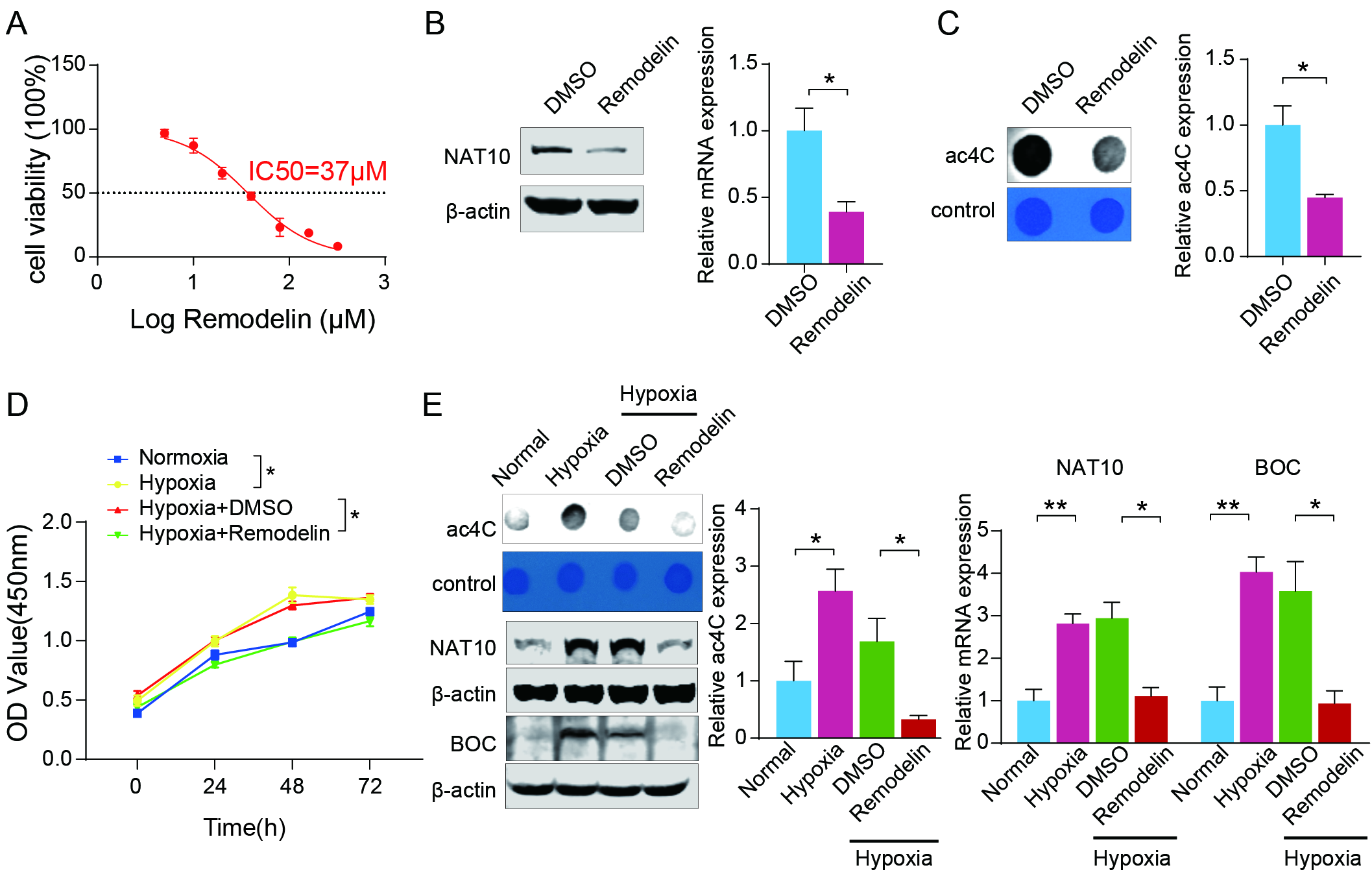

Supplement: Supplementary file 10 — FigureS8 [file 41419_2025_8315_MOESM10_ESM.tif]
